# Cross-Species Transcriptomics Analysis Highlights Conserved Molecular Responses to Per- and Polyfluoroalkyl Substances

**DOI:** 10.3390/toxics11070567

**Published:** 2023-06-29

**Authors:** Livia Beccacece, Filippo Costa, Jennifer Paola Pascali, Federico Manuel Giorgi

**Affiliations:** 1 Department of Pharmacy and Biotechnology, University of Bologna, 40126 Bologna, Italy; livia.beccacece2@unibo.it (L.B.); filippo.costa9@studio.unibo.it (F.C.); 2 Department of Cardiac, Thoracic, Vascular Sciences and Public Health, University of Padua, 35121 Padua, Italy; jennifer.pascali@unipd.it

**Keywords:** PFAS, metabolism, transcriptomics, transcriptome, cross-species correlation

## Abstract

In recent decades, per- and polyfluoroalkyl substances (PFASs) have garnered widespread public attention due to their persistence in the environment and detrimental effects on the health of living organisms, spurring the generation of several transcriptome-centered investigations to understand the biological basis of their mechanism. In this study, we collected 2144 publicly available samples from seven distinct animal species to examine the molecular responses to PFAS exposure and to determine if there are conserved responses. Our comparative transcriptional analysis revealed that exposure to PFAS is conserved across different tissues, molecules and species. We identified and reported several genes exhibiting consistent and evolutionarily conserved transcriptional response to PFASs, such as *ESR1*, *HADHA* and *ID1*, as well as several pathways including lipid metabolism, immune response and hormone pathways. This study provides the first evidence that distinct PFAS molecules induce comparable transcriptional changes and affect the same metabolic processes across inter-species borders. Our findings have significant implications for understanding the impact of PFAS exposure on living organisms and the environment. We believe that this study offers a novel perspective on the molecular responses to PFAS exposure and provides a foundation for future research into developing strategies for mitigating the detrimental effects of these substances in the ecosystem.

## 1. Introduction

Per- and polyfluoroalkyl substances (PFASs) are a heterogeneous class of fluorinated synthetic compounds encompassing a great number of molecules with different structures [1]. They have gained global notoriety due to their persistence and adverse effects on living organisms and environmental health [2]. While a compendious definition of these chemicals is challenging to provide, the Organization of Economic Co-operation and Development (OECD) recently defined PFAS as molecules containing at least a perfluorinated methyl (–CF_3_) or a perfluorinated methylene group (–CF_2_–) without any H/Cl/Br/I attached to it [3]. However, there are several PFAS classifications that are based on diverse definitions and include a variable number of molecules. For instance, PubChem’s classification, based on OECD’s general description, includes more than 6.3 million PFAS molecules [4], while the United States Environmental Protection Agency’s (EPA) classification, founded on molecular substructures and a threshold of fluorine percentage [5], contains 14,735 compounds [6]. Despite the challenges and discrepancies in defining these substances, the OECD currently recognizes 4730 molecules as bona fide PFASs, which are further classified based on their carbon chain length and molecular structure, which determines their unique physicochemical properties and environmental behavior. Short-chain and long-chain PFASs are distinguished based on their carbon chain length, and polymeric and non-polymeric PFASs are differentiated based on the presence or absence of repeating monomer units in their molecular structure. Moreover, PFASs are commonly classified based on their legal status as either legacy or emerging PFASs. Emerging PFASs are compounds such as HFPO-DA or GenX, ADONA, or C6O4, which were introduced after the ban on perfluorooctane sulfonate (PFOS) and perfluorooctanoate (PFOA) production, import, and use. These emerging PFASs are characterized by a shorter C-F backbone and are considered less hazardous than legacy PFASs due to their lower bioaccumulation potential and toxicity [7].

PFASs have unique chemical properties that fostered their widespread production and use in a multitude of industrial products since the 1950s [8]. The C–F bond in PFAS molecules confers high molecular stability but also results in high resistance to degradation [8]. Additionally, the chemical attributes of amphiphilic and hydrophobic PFASs make them ideal surfactants and surface protectors, while also making them resistant to high temperatures. The versatility of PFASs has led to their use in a wide range of products, including non-stick pans, firefighting foams (aqueous film-forming foams, AFFF), waterproof textiles, pesticides, building and construction materials, cleaning products and medical and personal care products, among many others [2,8].

Despite their widespread use, the potential risks of PFAS exposure to human health and the environment have become increasingly apparent. PFASs have been found to be ubiquitously present in the environment, where, thanks to their intrinsic chemical stability, they can persist for several years owing to their resistance to degradation [2]. Water basins have been identified as major repositories of PFASs and are capable of transferring these substances over long distances, making the water ecosystem a crucial gateway for PFAS entry into the food chain up to humans [9,10]. Numerous studies have focused on specific PFAS molecules, such as PFOS and PFOA, and have shown that their accumulation can have detrimental effects on aquatic and terrestrial ecosystems, as well as on animal species and plants. As a result, limitations on the use of PFOA and PFOS were introduced in some regions [2,11,12,13,14]. Moreover, the presence of PFASs in human biological matrices has been highlighted in numerous studies, with a global distribution. PFASs have been detected in serum [15,16], breast milk [17,18], placenta [19,20], hair [21] and semen [22], indicating widespread exposure in human populations.

The vast majority of physiological and molecular research on PFASs has been directed towards human health, revealing their toxicological effects on biological processes and metabolism. These negative impacts include reduced fertility, altered gene transcription [12,23,24,25,26,27,28,29] and the promotion of certain types of cancer, such as kidney and liver cancer [30,31]. However, there are conflicting data on the involvement of PFASs in cancer pathogenesis [30]. Furthermore, PFASs have been shown to negatively affect the activity of the immune system, particularly in children, by impairing immune reactions and vaccination responses [23,24,25]. Lipid metabolism is also heavily impacted by PFAS exposure, leading to dyslipidemia and increased plasma levels of cholesterol [32,33,34,35,36,37].

Numerous studies have demonstrated that PFASs affect multiple species through detectable molecular mechanisms [38,39,40,41]. These compounds can directly interact with molecules such as the peroxisome proliferator-activated receptor α (PPARα), which mediates PFAS toxicity [42]. Most importantly, PFASs are capable of modifying the transcriptional expression of many genes in humans and other species [12], which has significant repercussions on the mentioned pathways and diseases.

Despite the vast evidence of transcriptional changes induced by PFASs in multiple species and despite the presence of numerous quantitative transcriptome-wide studies measuring gene expression responses to PFAS exposure [38,39,43], a comprehensive and comparative analysis of the data generated by these studies has yet to be performed. To address this gap, we propose a rational integration and comparison of transcriptome-wide studies performed in animal species and cell models, in the form of RNA-Seq or microarray datasets. Using the opportunities offered by transcriptomics, we aim to elucidate the molecular effects induced by PFASs not only at the single gene level but also across different pathways and cell types. Our research provides a comprehensive understanding of the molecular mechanisms underlying PFAS toxicity that translate across species while accelerating evidence-based policies and treatments to safeguard public and environmental health.

## 2. Materials and Methods

### 2.1. Data collection and Processing

We conducted an extensive literature search across databases to identify all transcriptome-wide quantitative studies focusing on the effects of PFASs on animal samples. A total of 11 transcriptomics datasets were identified, containing publicly available data from 7 different species (*Homo sapiens*, *Mus musculus*, *Caenorhabditis elegans*, *Danio rerio*, *Gadus morhua*, *Micropterus salmoides*, *Pimephales promelas*) (Table 1) [38,39,40,41,43,44,45,46,47,48,49]. Raw data associated with these studies were retrieved from the Gene Expression Omnibus (GEO) database (https://www.ncbi.nlm.nih.gov/geo/ accessed on 1 December 2022) [50] and the Sequence Read Archive (SRA) database (https://www.ncbi.nlm.nih.gov/sra accessed on 1 December 2022) [51], both hosted at the National Center for Biotechnology Information (NCBI).

Raw sequence data (FASTQ files) from the *D. rerio* [41] and *G. morhua* [43] datasets were downloaded from the SRA database [51] using SRA Toolkit version 3.0.1. These reads were aligned to the respective reference genomes (zebrafish genome version danRer11/GRCz11 and Atlantic cod genome version gadMor3.0) using the HISAT2 alignment program version 2.1.0 [52]. The BAM files containing the aligned reads of zebrafish and Atlantic cod were processed with featureCounts version 2.0.0 [53] to obtain matrices containing the gene counts for each sample. The other datasets were directly downloaded from the NCBI GEO database [50] with most of them being in the form of gene counts matrices, while the Pfohl et al. 2021 dataset [47] was available through CEL files (which are files commonly produced by Affymetrix DNA microarray image analysis software).

All statistical analyses were conducted in the R statistical software version 4.2.2 and Bioconductor version 3.16. To generate graphs for this manuscript, we used base R functions and R packages including *ggplot2* version 3.4.1 [54], *corrplot* version 0.92, *corto* version 1.2.0 [55] and *ComplexHeatmap* version 2.14.0 [56].

RNA-Seq gene-based reads counts were directly loaded into the R environment, while R package *oligo* version 1.62.2 was used to import and process CEL files. Microarray data were normalized using RMA normalization [57]. R package *GEOquery* version 2.66.0 [58] was utilized to recover the metadata containing the information about the experimental design.

All sequencing data alignment and gene expression quantification steps were performed on an HPC-dedicated DELL EMC server with an AMD EPYC 7301 32-core processor and 256 GB of RAM. Microarray normalization, post-normalization statistical analysis and graphics were carried out on a Windows 10 machine Intel Core i7-10700 CPU with 32 GB of RAM (manufacturer: LENOVO, Beijing, China).

### 2.2. Differential Gene Expression Analysis

To comprehensively assess the transcriptome-wide response to PFASs, we designed an approach of comparison of 110 total differential gene expression contrasts, using for each dataset a balanced PFAS-treated vs. control design, with at least three replicates per group. For RNA-seq data, we used the *DESeq2* R package version 1.38.3 [59] on raw read counts. For microarray data, we implemented the default pipeline of the *limma* R package version 3.54.1 [60]. Due to the significantly higher number of contrasts in two *H. sapiens* datasets [38,44] than all others (Table 1), we decided to retain only a PFAS concentration of 20 µM in these two datasets [38,44]. In the case of the *P. promelas* dataset [48], low-exposure specimens from Upper Prior Lake were used as PFAS-treated samples. Overall, the differential gene expression analysis was implemented on 110 separated contrasts, encompassing all datasets (Table 1). All contrasts yielded more than 10 significantly (at *p* ≤ 0.05) differentially expressed genes in response to PFASs (Supplementary Table S1).

For each contrast of the datasets, we generated a gene-by-gene transcriptome-wide *signature*, defined by the following formula:

−log_10_(*p*) × sign(log(FC))

where *p* represents the *p*-value of the differential expression (calculated by *limma* or *DESeq2*) and FC represents the fold change of the differential expression.

In essence, this formula (implemented in several other transcriptomics publications, such as Alvarez et al., 2016 [61]) assigns a numerical value to each gene that is positive for significantly up-regulated genes, and negative for significantly down-regulated genes. The magnitude of the numerical value is proportional to the tested significance of the change.

### 2.3. Ortholog Prediction

To enable the comparison of gene expression data across different species, we devised a phylogenetic gene conversion approach to convert all gene signatures to a common gene identifier.

In order to do so, we performed a direct species-to-human conversion using the DRSC Integrative Ortholog Prediction Tool (DIOPT) database version 9.0 [62] for all available species in the database. For species not available in DIOPT, we utilized the R package *orthogene* version 1.4.1 [63] to perform the conversion. In instances where species were not available in either database (specifically, for *Micropterus salmoides* and *Pimephales promelas*), we employed a bidirectional best-hit approach based on BLASTn version 2.12.0+ [64], using the sequences associated with each microarray probe as a query, and the zebrafish cDNA version danRer11/GRCz11 as the target database. We then converted the identifiers from zebrafish to human using DIOPT. All ortholog conversions used in this study are available in Supplementary Table S2.

The resulting matrix of signatures, based on the most likely human ortholog, contained 110 contrasts (PFAS vs. control) and was used for subsequent analysis (Supplementary Table S3).

### 2.4. Signature Analysis

To assess the similarities between gene expression signatures, we employed Pearson correlation, provided by the R basic function cor().

For the pathway enrichment analysis, we retrieved gene sets from KEGG, WikiPathways and Gene Ontology using the Molecular Signatures Database (MSigDB) [65]. We accessed the database via the R package *msigdbr* version 7.5.1 and implemented the enrichment analysis on the signatures using the R package *fgsea* version 1.24.0. This package uses an algorithm for expedited and parallel gene set enrichment analysis [66].

To integrate the normalized enrichment scores (NES) derived from the pathway enrichment analysis, we employed Stouffer integration as implemented by the *corto* R package version 1.2.0 [55] and as performed before [61]. Z-scores were converted to *p*-values, where needed, using the z2p() function from the aforementioned *corto* package [55]. All *p*-values were corrected using the Benjamini–Hochberg method. All the R code used to integrate data and generate the figures in this paper is available on Github at the following address: https://github.com/federicogiorgi/pfas (accessed on 14 June 2023).

### 2.5. Metabolites Prediction

We employed a correlation-based method to predict metabolites based on gene expression signatures, as described in Cavicchioli et al., 2022 [67]. Briefly, this method assesses the correlation structure between metabolites and transcripts measured in the Cancer Cell Line Encyclopedia metabolomics/transcriptomics dataset [68] and then predicts the metabolite levels in scenarios where only transcripts are available.

We applied this analysis to 55 PFAS exposure contrasts of three human datasets included in this study [38,44,45]. Prior to the analysis, RNA-seq gene expression count data were normalized using variance stabilizing transformation (VST) [69]. We then integrated the normalized enrichment scores (NESs) generated contrast by contrast (Supplementary Table S4) using the Stouffer integration method, as implemented by the *corto* R package [55]. The *p*-values were corrected using Benjamini–Hochberg method.

## 3. Results

### 3.1. Datasets

Using the literature and biological databases, we searched all publicly available transcriptome-wide PFAS quantitative data, in order to build the most comprehensive collection available to date. Our search retrieved 2144 samples from 11 datasets and from 7 different species for our analysis. Table 1 provides detailed information about each dataset, including the overall study design, tested PFAS molecules, number of samples, and tissues analyzed.

### 3.2. Correlation Analysis

**Figure 1:**
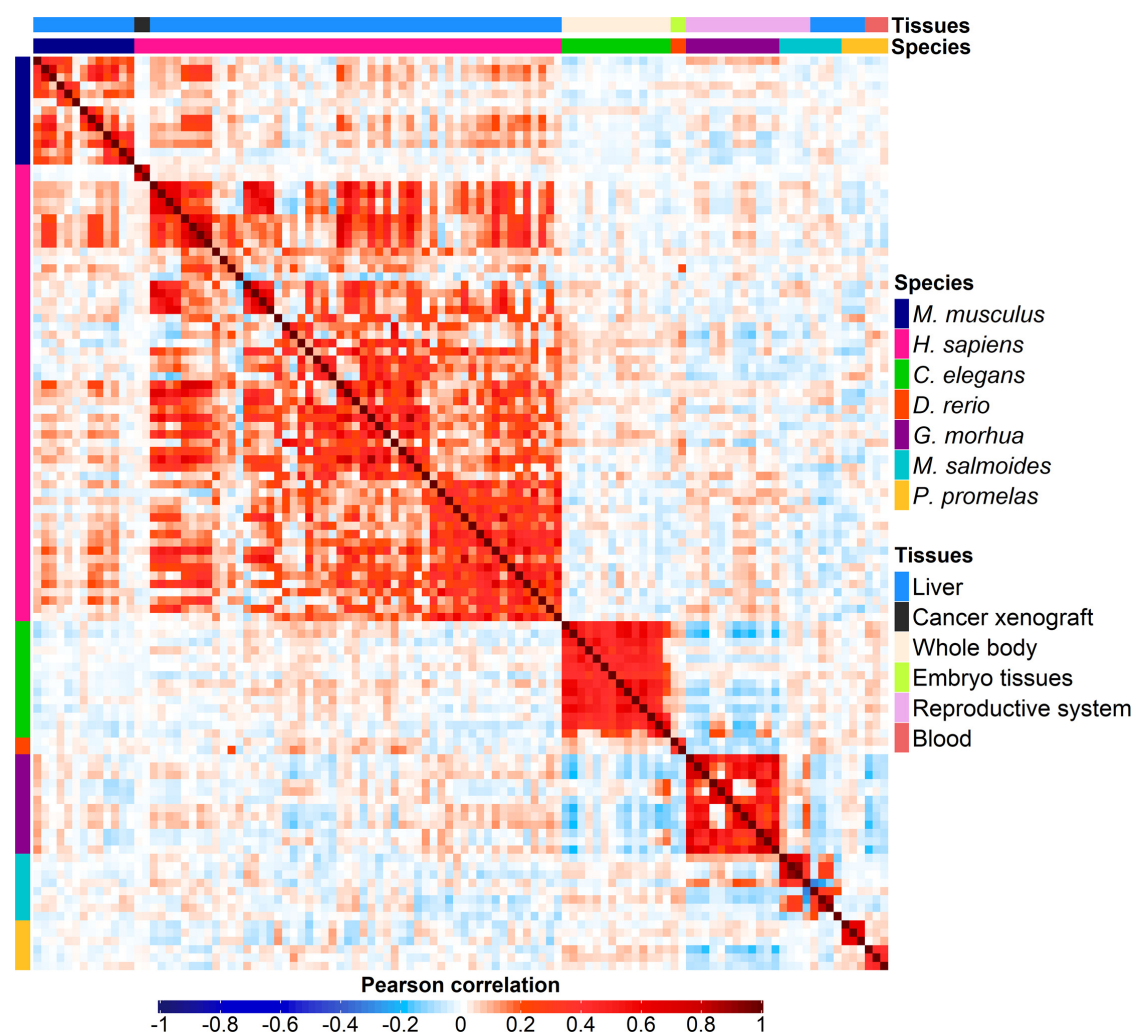
Heatmap displaying the correlation among 110 different PFASs vs. control differential expression contrasts. The color gradient ranges from blue (denoting negative correlation) to red (denoting positive correlation), with darker colors indicating higher correlation values. Each colored dot indicates the correlation value between any two contrasts of the final signature matrix (Supplementary Table S3). The upper bar denotes the tissue of origin of each contrast.

To assess whether PFASs promote similar responses across species, we extracted transcriptional signatures from each PFAS vs. control contrast (Supplementary Table S3). Our comparative transcriptional analysis revealed that exposure to different PFAS molecules determines both intra- and interspecies correlations (Figure 1), indicating that this class of compounds induces conserved biological responses among species, despite the high phylogenetic distance between the species analyzed herein. Notably, our analysis demonstrated a general preponderance of positive correlation, with greater values in intraspecies comparison (Figure 1 and Figure S1).

**Figure 2:**
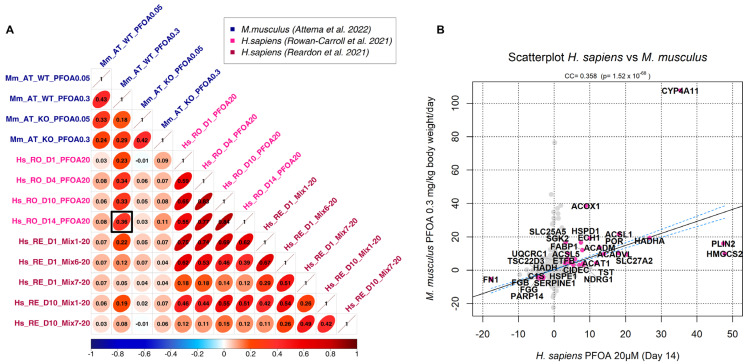
(**A**) Correlation plot of *M. musculus* and *H. sapiens* exposed to the PFOA molecule [38,39,44]. As an example, the greatest correlation achieved between human and mouse contrasts (0.36) is highlighted by a black box. (**B**) Scatterplot showing the correlation among the contrasts of mouse and human highlighted by a black box in the previous plot. The highlighted genes are the most significant genes driving the correlation between the two species, defined by significant transcriptional change (*p* ≤ 0.001) in response to PFAS exposure in both species.

Relating to cross-species correlation, our analysis revealed a strong positive correlation between the transcriptional signatures of *H. sapiens* and *M. musculus*, especially when exposed to the same PFAS molecule (Figure 2A and Figure S2), highlighting the close evolutionary proximity between the two species. We detected interspecies positive correlations as high as 0.36 (Figure 2A), which is extremely significant (*p*-value = 1.52 × 10^−68^, Figure 2B). This similarity was observed between the liver of wildtype mice [39] and human liver spheroids [38], both exposed to PFOA, although at different concentrations and exposure times.

This correlation between the transcriptional signature of *H. sapiens* [38] and *M. musculus* [39] is driven by genes that are differentially expressed (*p*-value ≤ 0.001) in both species in response to PFAS exposure, as highlighted in Figure 2B. Among these genes, *CYP4A11* is highly up-regulated in both species and encodes an ω-hydroxylase of the CYP450 gene family, which is involved in the metabolism of fatty acids, such as arachidonic acid. *CYP4A11* is highly expressed in the liver and kidney, where it synthetizes the 20-hydroxyeicosatetraenoic acid (20-HETE) from arachidonic acid [70]. 20-HETE has been shown to have cardiotoxic and vasoconstrictive activity, and its increased synthesis is associated with vascular inflammation and hypertension [71]. Remarkably, *CYP4A11* up-regulation has been associated with non-alcoholic fatty liver disease (NAFLD), since it increases the intracellular production of reactive oxygen species (ROS) and pro-inflammatory cytokines [72]. Our result is in line with data showing that exposure to PFOA is positively related to NAFLD development [73]. The other up-regulated genes (Figure 2B) are mainly implicated in lipid metabolism, mitochondrial function, and stress response, while down-regulated genes participate in immune response and inflammation, thrombosis, and cellular adhesion.

Our analysis also revealed a significant positive correlation (0.22, *p*-value = 7.89 × 10^−20^) between the transcriptional signatures of *H. sapiens* from Rowan-Carroll et al.’s (2021) dataset [38] and *D. rerio* from Dasgupta et al.’s (2020) dataset [41]. Notably, this correlation is driven by the down-regulation of various genes encoding different types of collagen (Supplementary Figure S3).

**Figure 3:**
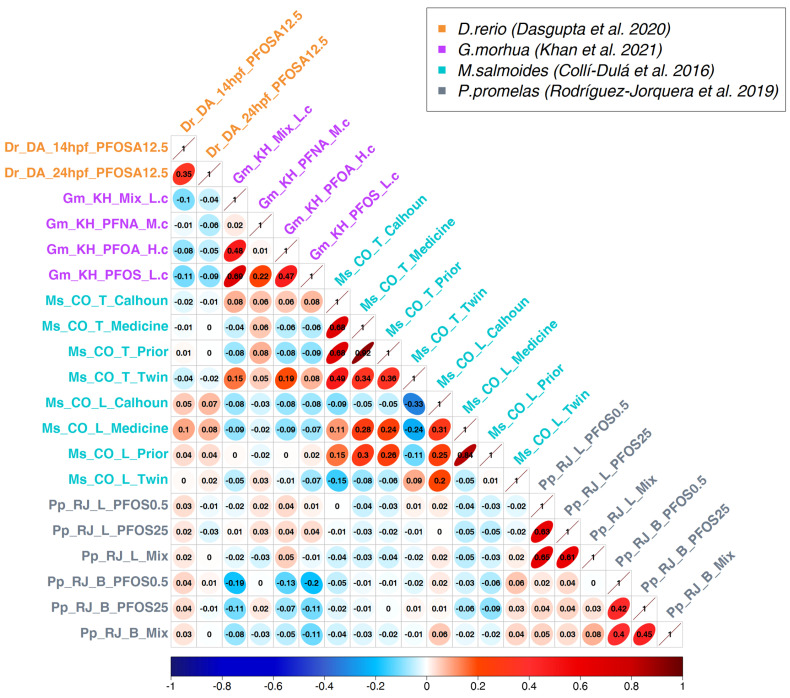
Comparison between PFAS exposure signatures in four different fish species [41,43,48,49]. For the Atlantic cod (*Gadus morhua*) dataset, one concentration for each PFAS molecule was selected. The full analysis including all concentrations and contrasts is displayed in Supplementary Figure S4.

In addition to positive correlations, our analysis also highlighted significant negative correlations, both between distinct species and between different tissues of the same species (Figure 1 and Figure S1). We hypothesize that exposure to PFAS substances elicits opposite responses depending on the tissue analyzed, both within and across different species. These results might be due to histological differences in gene expression among distinct tissues, as similarly observed by Glinos and colleagues [74], where the same molecules trigger distinct transcriptional changes as demonstrated for drug-metabolizing enzymes [75]. Illustratively, the negative values were most prominently observed in fish species, where different tissues of distinct species, such as *G. morhua* (ovary [43]) and *P. promelas* (blood [49]), and of the same species, as in the case of *M. salmoides* (liver and testis [48]), exhibited moderate but significant negative correlations (Figure 3 and Figure S4). For instance, the negative correlation of −0.2 between the blood sample of *P. promelas* exposed to PFOS at 0.5 µg/L and the ovary of *G. morhua* exposed to PFOS at low concentration is particularly significant with a *p*-value of 6.99 × 10^−49^.

### 3.3. Generation of a Cross-Species PFAS Responses

Once it was ascertained that exposure to PFAS molecules induces significantly similar transcriptional changes across different species, our primary objective was to identify which genes are most responsible for this transcriptional conservation and therefore define the molecular basis for this observed conservation. In order to overcome the uneven representation of species in our signature analysis (Table 1), we performed a weighted Stouffer integration on the signature matrix, giving equal representation to each species in our dataset. This approach enabled us to pinpoint the genes that were over- and under-expressed across all species.

**Figure 4:**
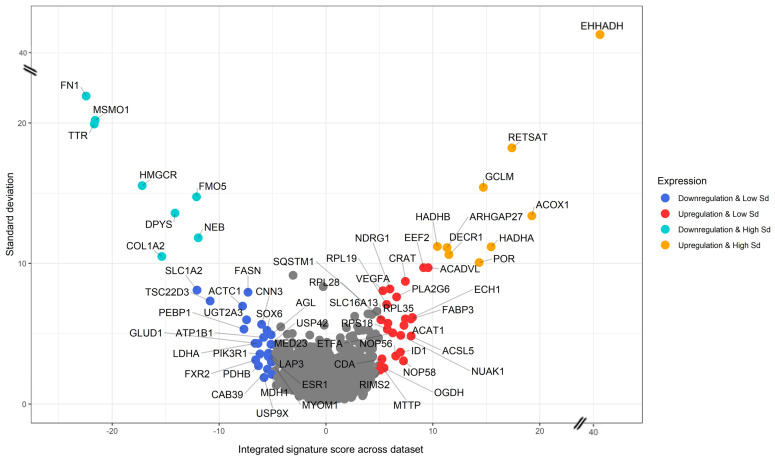
Plot showing the integrated response to PFASs across 110 contrasts. Each point represents a gene. The *x*-axis indicates the integrated signature value (obtained by integrating signatures across the dataset using the Stouffer method). The *y*-axis indicates the standard deviation of the signature across the dataset. In red and orange, genes with the highest positive integrated signature (i.e., conserved PFAS-induced up-regulation across species), in blue and cyan, genes with the highest negative integrated signature (i.e., conserved PFAS-induced down-regulation across species). Genes in orange or cyan are also characterized by signature standard deviation above 10, indicating heavier fluctuations across the dataset (see also Figure 5 and Figure S5).

We successfully identified 3435 genes appearing in at least six species of the seven species included in our dataset (Figure 4). Our analysis highlights genes that are most consistently up- or down-regulated by PFASs in the dataset. Nine genes (*EHHADH*, *RETSAT*, *GCLM*, *ACOX1*, *HADHB*, *ARHGAP27*, *DECR1*, *HADHA*, and *POR*, depicted in orange in Figure 4) are characterized by an elevated and positive integrated signature (≥10 Stouffer integrated Z-score, corresponding to *p*-value ≤ 1.6 × 10^−23^), but also by a high (≥10) signature standard deviation across our dataset; these nine genes are therefore induced by PFASs in a strong and conserved way, albeit with heavy fluctuations across contrasts (see also Supplementary Figure S5), which may indicate outlying contrasts. We then highlighted 25 genes significantly up-regulated (≥5 Stouffer integrated Z-score, corresponding to *p*-value ≤ 5.8 × 10^−7^) with lower standard deviation (<10), highlighted in red in Figure 4, and including acetyl-CoA acetyltransferase 1 (*ACAT1*), an inhibitor of DNA binding 1 (ID1) and vascular endothelial growth factor A (*VEGFA*). Among genes consistently repressed by PFASs, we found eight genes (*FN1*, *MSMO1*, *TTR*, *HMGCR*, *FMO5*, *NEB*, *DPYS*, and *COL1A2*, indicated in cyan in Figure 4) with strong down-regulation across the dataset (≤ −10 Stouffer integrated Z-score, corresponding to *p*-value ≤ 1.6 × 10^−23^) and high standard deviation. We also highlighted 23 genes down-regulated at a lower standard deviation (<10) with ≤ −5 Stouffer integrated Z-score, corresponding to *p*-value ≤ 5.8 × 10^−7^, which include the PFAS-repressed oncogene *ESR1*, encoding for estrogen receptor.

**Figure 5:**
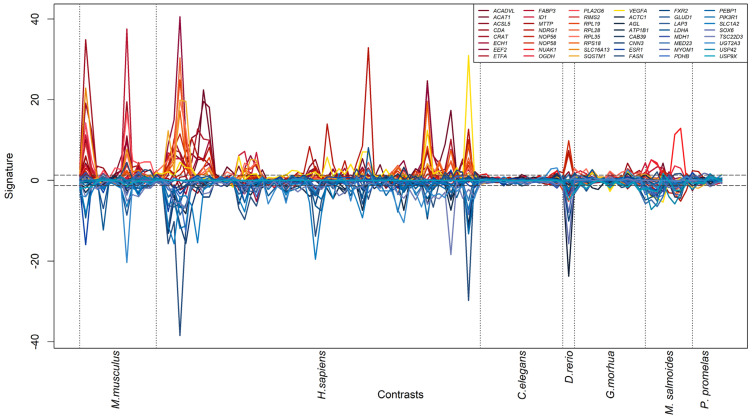
Line graph indicating the levels of expression of selected genes in response to PFAS molecules in different species. Each line is one gene: the genes shown here are the most consistently up- or down-regulated with low standard deviation, as extracted from the red and blue points of Figure 4. The *x*-axis reports all the 110 contrasts analyzed in the integrated dataset, grouped by species. The *y*-axis reports the signature for each gene, representing the significance (and sign) of the gene’s transcriptional response to PFASs. The horizontal lines delimit the *p*-value thresholds of 0.05.

While useful as a summarization technique, signature integration may hide odd behaviors in the response to PFASs across different contrasts. In order to investigate this potential issue, we visualized the signature of each of the 48 genes (25 + 23) up- and down-regulated by PFASs across the 7 species and 110 contrasts (Figure 5). All prioritized genes indeed show a consistent pattern of activation. It is to be noted, however, that for the data deriving from two species, the response to PFASs is almost negligible (*C. elegans* and *P. promelas*). Genes with higher standard deviation (cyan and orange dots in Figure 4) also showed consistent response to PFASs; however, their scores were heavily dominated by specific contrasts in *M. musculus* and *H. sapiens* (Supplementary Figure S5).

The 65 genes prioritized by our analysis were found to be consistently differentially expressed not only across different species but also across different tissues. A more detailed analysis of the signatures shows that the strongest impact of PFASs is observed in the liver and reproductive system of *M. musculus, H. sapiens*, *G. morhua* and *M. salmoides*, together with a strong response to PFASs in the embryonal development of *D. rerio*.

A closer analysis of the genes most affected by PFASs across species (Figure 4 and Figure 5) shows a noticeable prevalence of certain biological pathways, most notably lipid metabolism (*HADHA*, *HADHB*, *ACOX1*, *ACSL5*, *FABP3*, *CRAT*, *PLA2G6*), hormone-associated signal transduction (*NDRG1*, *ESR1*, *PIK3R1*, *SQSTM1*, *TSC22D3*), pyrimidine metabolism (*DPYS*, *CDA*), also with a relevant presence of mitochondrial (*CRAT*, *DECR1*, *GLUD1*, *HADHA*, *HADHB*, *PDHB*) and peroxisomal (*ACOX1*, *CRAT*, *ECH1*) genes. The presence of so many genes involved in lipid metabolism confirms previous data demonstrating that this metabolic process is highly affected by PFAS exposure [32,33,34,35,36]. A peculiar finding here is the *USP42* gene, which is down-regulated by PFASs across species (Figure 4). *USP42* encodes a deubiquitinating enzyme involved in embryonal testis development and spermatogenesis [76], and its presence amongst the most consistently down-regulated genes may provide a molecular link to the previously observed effects of PFASs on the male reproductive system [77].

### 3.4. Pathway Enrichment Analysis

**Figure 6:**
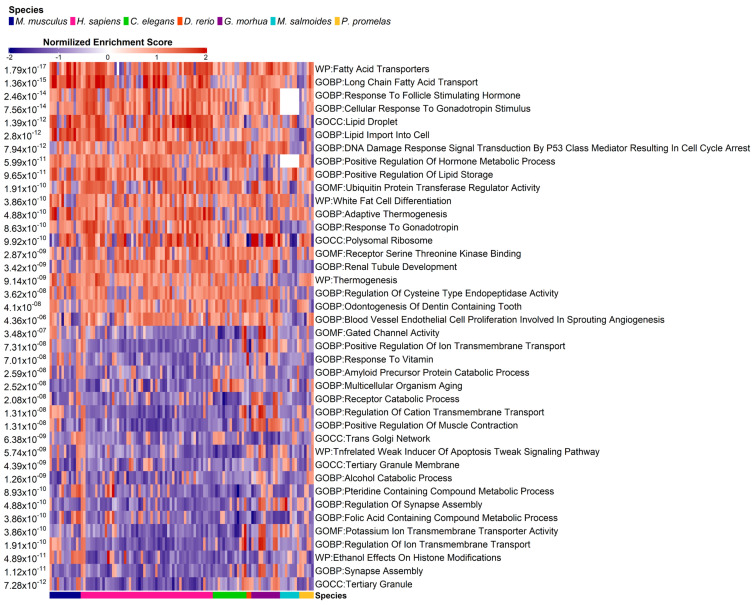
Heatmap showing the 20 most significant pathways that are up-regulated and down-regulated across species. The blue-red color scale is proportional to the strength of the calculated pathway NES. White cells indicate contrasts with insufficient (<5) pathway genes to reliably calculate GSEA. The bottom bar indicates the species of each contrast in color code. The *p*-adjusted on the left side indicates the integrated *p*-value of pathway enrichment calculated across species.

In order to perform a more rigorous investigation of the molecular and biological processes most affected by PFASs, we calculated the pathway enrichment contrast of the signature matrix (Supplementary Table S3) using the GSEA algorithm [78]. We then integrated the normalized enrichment scores (NES) across the datasets to identify the pathways that were predominantly up- and down-regulated. We identified 3275 pathways significantly up- and down-regulated by PFASs across species (integrated *p*-adjusted ≤ 0.05). In Figure 6, we show the most significantly up- and down-regulated pathways.

As inferred in the previous paragraph, lipid metabolism appears to be amongst the cellular component most up-regulated in response to PFASs (Figure 6), with the “fatty acid transporters” WikiPathways gene set characterized by a *p*-adjusted of 1.79 × 10^−17^ and the Gene Ontology “lipid import to cell” gene set at *p*-adjusted = 2.80 × 10^−12^. As previously mentioned, PFASs have a significant impact on this metabolic process, for example through the induction of dyslipidemia, characterized by elevated total cholesterol plasma levels [32,33,34,35,36,37], and NAFLD [73,79], characterized by fat accumulation in the liver that leads to impaired organ function. It is important to note that children and adolescents are equally susceptible to the effects of PFAS exposure on lipid metabolism [80], as studies have reported that this group is at a higher risk of developing nonalcoholic steatohepatitis (NASH) and NAFLD [81]. A significant body of research has confirmed this effect of PFAS on lipid metabolism in human [32,33,34,35,36], mouse [37], and zebrafish [82] with comparable lipid changes observed across species. Strikingly, among the up-regulated pathways, there are some that relate to the response to gonadotropins (Gene Ontology “Cellular response to gonadotropin stimulus”, *p*-adjusted 7.56 × 10^−14^) and to FSH (follicle-stimulating hormone, represented by Gene Ontology term “Response to FSH” at *p*-adjusted 7.56 × 10^−14^). These hormones stimulate the development and growth of the ovarian follicles, thereby affecting fertility [83]. Previous data have shown that PFAS molecules directly influence the secretion of gonadotropin-releasing hormone (GnRH), in turn promoting the expression of gonadotropins, depending on the dose and period of exposure [84].

The most significant down-regulated pathway is represented by the Gene Ontology “Tertiary granule” gene set (adjusted *p*-value = 7.28 × 10^−12^). Tertiary granules are secretory granules of neutrophil cells that contain extracellular-matrix-degrading enzymes and are implicated in the inflammatory response [85]. This result highlights a possible mechanism for the immunotoxicity deriving from PFAS exposure [23,24,25].

In summary, the identified pathways underscore the complex and diverse nature of PFAS toxicity, with significant implications for lipid metabolism, immune response, and reproductive function.

### 3.5. Prediction of Affected Metabolites

**Figure 7:**
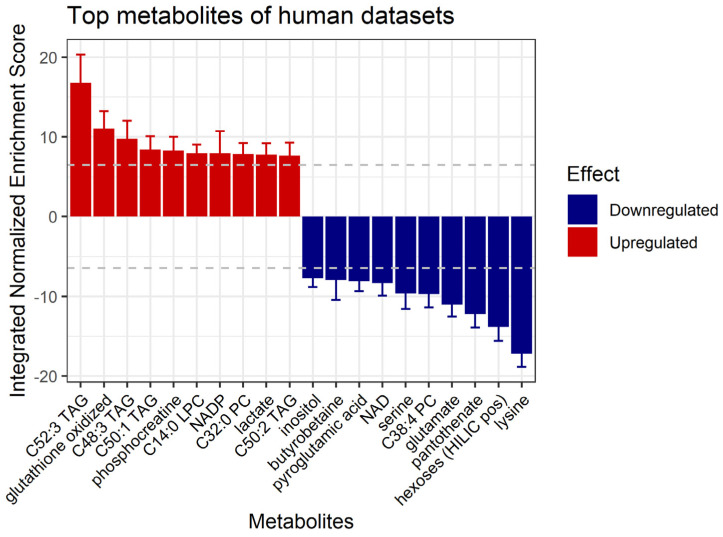
Bar plot indicating the integrated normalized enrichment score of predicted metabolic changes upon exposure to PFASs in all human contrasts. TAG: triacylglycerol. LPC: lysophosphatidylcholine. PC: phosphatidylcholine. NAD: nicotinamide adenine dinucleotide.

As the last step of our analysis, we wanted to test the possibilities provided by a newly developed algorithm to infer metabolite differential abundance from gene expression data [67], based on correlation data from the largest (in terms of samples) transcriptomics/metabolomics dataset available to date, the human Cancer Cell Line Encyclopedia dataset [68]. As the method has been developed and tested only on human data, we decided to test it on all human contrasts, generating a predicted normalized NES in response to PFASs for 147 metabolites across all human PFAS vs. control comparisons (Supplementary Table S4). We then proceeded to integrate all the PFAS-related NESs across contrasts, to provide a human-specific prediction of metabolite response to PFASs. The top 20 most significantly up- and down-regulated metabolites are displayed in Figure 7.

Our analysis shows that exposure to different PFAS compounds stimulates the dysregulation of different types of lipids (triacylglycerols, phosphatidylcholines and lysophosphatidylcholines), amino acids, vitamins and coenzymes. Amongst the most up-regulated lipids, we found triacylglycerol C52:3, which is predicted to have the highest induction via PFASs (NES = 16.78, *p*-adjusted = 1.72 × 10^−62^). Another compound highly up-regulated by PFASs is oxidized glutathione, with NES = 11.07 (*p*-adjusted = 6.95 × 10^−28^).

In contrast, among the top 10 most down-regulated metabolites, we found 3 amino acids: lysine (NES = −17.21, *p*-adjusted = 4.11 × 10^−65^), glutamate (NES = −11.05, *p*-adjusted = 6.95 × 10^−28^) and serine (NES = −9.65, *p*-adjusted = 1.12 × 10^−21^).

## 4. Discussion

Our comprehensive analysis gathered and compared all currently available quantitative transcriptomics datasets on PFAS response in animals. The resulting data collection is heterogeneous in terms of species, compounds, concentration, time of exposure, organ and sequencing technology. However, despite this biological diversity, we detected significant recurring responses both at the gene and pathway levels, indicating a cross-compound, cross-tissue and cross-species conservation of transcriptional effects induced by PFASs.

The first important result is that there is detectable and significant cross-species transcriptomics similarity in the response to PFASs (Figure 1 and Figure S1), with higher similarity between closer species (Figure 2 and Figure 3 and Figures S1, S2 and S4). However, some transcriptional effects induced by PFASs are conserved even in species as distantly related as human and zebrafish (Supplementary Figure S3).

Our investigation then deepened towards specific genes and pathways underlying this cross-species conservation. For example, our analysis detected a strongly conserved PFAS-induced up-regulation of lipid metabolism and transport, as well as gonadotropin and FSH pathways (Figure 6). All these processes are clearly related to ovarian development, estrogen production, ovulation and the physiological functioning of the female reproductive system [86], and this deregulation may provide molecular mechanisms to explain PFAS-related detrimental effects on fertility [26,27,28,29,83] and fetal development [87,88,89,90,91].

Another interesting finding is the conserved down-regulation of another component of ovarian development, the *ESR1* gene (Figure 4 and Figure S6). *ESR1* encodes for the estrogen receptor alpha (ER-α), a nuclear receptor that influences the expression of numerous genes and physiological processes [92]. By interacting with estrogens, mainly with estradiol (E2), it affects female fertility being essential for ovulation, cellular proliferation and tissue differentiation [92]. Ovary E2/ER-α axis promotes ovulation, and a lower or absent expression of ER-α is associated with infertility [92,93]. ER-α is expressed even in kisspeptin neurons, in which the E2-ER-α interaction inhibits the activity of these neurons and the subsequent synthesis of gonadotropins in the hypothalamic-pituitary axis [94,95]. A lack of ER-α is also associated with increased synthesis of gonadotropins [96], which in turn determines the production of estradiol in the ovary [83]. *ESR1* down-regulation is associated with the up-regulation of response to gonadotropins also in polycystic ovary syndrome, leading to infertility [96]. Previous studies have already shown reduced *ESR1* expression and transcriptional regulatory activity in mice and humans [37,97] in response to PFAS exposure, giving further validation to our data.

There also appear to be effects of PFASs that go beyond the disruption of reproductive functionality. For example, our data show the up-regulation of the *ID1* gene across species (Figure 4 and Figure 5). *ID1* encodes for an inhibitor of DNA-binding proteins, which regulates the cell cycle and differentiation. The overexpression of *ID1* has been linked to various types of cancer, including leukemia, breast and pancreatic cancer [98,99]. Epidemiologic data suggest that PFASs are also associated with certain types of cancer, with some elements suggesting a pro-oncogenic effect [30]. Notably, elevated exposure to PFOA and PFOS appears to significantly increase the mortality of individuals affected by liver cancer and malignant neoplasms of lymphatic and hematopoietic tissues [31]. The finding of a conserved up-regulation of *ID1* may provide molecular support to the involvement of PFAS molecules in cancer pathogenesis.

Our integrated pipeline also detected a strongly conserved down-regulation of the tertiary granule pathway (Figure 6), a component of the immune defense against microorganisms enacted by neutrophil cells [85]. Recent independent findings also suggest that PFASs affect the function of neutrophils, likely inhibiting the formation of the granules or the degranulation process [100]. More scientific literature supports the fact that PFAS exposure impairs immune reactions, antibody production and vaccination responses, particularly in children exposed to PFASs during prenatal and postnatal periods [23,24,25]. This immunotoxicity has been observed not only in humans but also in other animals [23,24,25] and can increase the incidence and severity of many pathologies, including COVID-19 [101,102,103]. In addition, PFAS exposure increases the serum concentration of inflammatory and oxidative stress markers, potentially promoting the development of systemic diseases such as liver injury and cardiovascular diseases, including atherosclerosis and thromboembolic events [104,105,106]. The size and width of our collected PFAS transcriptomics dataset provide the neutrophil tertiary granule mechanism as a strong molecular candidate behind the observed toxic effect of PFASs on the immune system.

Our analysis shows that the transcription of genes involved in lipid metabolism is significantly affected by PFAS exposure, not only in humans but also in other species (Figure 4, Figure 5 and Figure 6). This is confirmed by previous studies, where PFAS exposure is associated with chronic dyslipidemia and increasing in lipid serum levels [32,33,34,35,36,37]. PFASs also increase the plasma levels of total cholesterol and triglycerides in a dosage-dependent manner [32,33,34,35,36]. It is worth noting that dyslipidemic changes are more pronounced in females than males [35,36] and are also observed in mice [37], as confirmed by our data. The relationship between dyslipidemia and PFASs has also been found in human children and adolescents [80], where exposure to these chemicals increases the risk of developing NASH and NAFLD [81] as well as impairing glucose metabolism [107]. Notably, we found that *CYP4A11*, previously associated with NAFLD [72,73], is highly up-regulated in both humans and mice, possibly indicating a causative role in NASH development due to PFAS exposure. The impact of PFAS on children is a crucial issue, and it seems that these chemicals can even be transferred through breastfeeding [17,18], which is of great concern.

Overall, our findings on the conserved pathway response to PFASs agree with the existing literature, especially concerning the disruption of lipid and energy metabolism [2,12]. While this validates our findings, it must be noted that the conservation of pathways and genes detected by our analysis is based on an animal-only dataset and, while we took measures to limit the preponderance of data from certain species (human and mouse), the available data are currently dominated by mammalians and vertebrates, with only one representative for invertebrates (*C. elegans*). If the future will provide more data for more species from different phylogenetic clades, it will certainly provide a more evolutionarily balanced overview of the conservation of transcriptional response to PFASs.

Using recent developments in gene expression data mining for metabolite level predictions [67], we could further analyze PFAS exposure through the prediction of their effects on the metabolome (Figure 7). In particular, the finding that PFAS molecules increase the levels of different kinds of lipids, mainly triacylglycerols as C52:3 TAG (Figure 7), is supported by studies in humans showing that PFAS exposure enhances the concentration of triglycerides and cholesterol in the blood [32,33,34,35,36]. Similarly, mice exposed to PFAS exhibit an increase in cholesterol and triglycerides in the liver [108]. Another PFAS-induced metabolite is oxidized glutathione, a thiol compound resulting from the reduction of reactive oxygen species, xenobiotics and drugs; it plays an important role in protection from oxidative stress and redox homeostasis maintenance, and its high levels are potentially toxic [109]. This induction is consistent with the previously shown PFAS-induced increase in glutathione S-transferase in the liver of Atlantic cod [110], whose increased activity is a marker of oxidative stress, and with reduced levels of reduced glutathione in human liver cells [111]. Our analysis predicted three amino acids amongst the top 10 metabolites down-regulated by PFASs: serine, lysine and glutamate (Figure 7). The down-regulation of serine agrees with the current literature displaying that serine deficiency is associated with an increase in lipid accumulation in the liver [112], a mechanism that mimics the impact of exposure to PFASs [12,47,79,108]. Lysine is an essential metabolite for a healthy pregnancy [113], and its deficiency is known to be detrimental to embryonal development [114], while glutamate is essential for embryonal neurogenesis [115]. Another metabolite predicted to be strongly down-regulated by PFAS is pantothenate (NES = −12.25, *p*-adjusted = 8.63 × 10^−34^), a vitamin required for the synthesis of coenzyme-A (CoA), which is in turn essential for fatty acid and energetic metabolism [116]. Pantothenate deficiency is associated with enhanced production of reactive oxygen species and oxidative stress [116], emulating the oxidative stress stimulated by PFAS exposure [117]. In the water flea *Daphnia magna*, pantothenate was experimentally shown to be down-regulated by PFAS exposure [118].

## 5. Conclusions

Our study constitutes the most extensive cross-species and cross-experiment analysis of transcriptional response to PFASs to date. With our collected dataset encompassing 7 species, 11 datasets, 110 contrasts and 2144 samples, we have demonstrated significant conservation of differential expression at both gene and pathway levels. Our analysis leverages the opportunities provided by contemporary transcriptome-wide quantitative technology and reveals a general disruption of hormonal synthesis and detection mechanisms, indicating that PFASs affect an ancient and conserved metabolic hormonal network, which has implications for several components of the ecosystem. While our work focused on commonalities between PFAS compounds, future studies, both computational and experimental, and fueled by the generation of more transcriptomics datasets, will certainly provide greater precision on the specific effects of different PFAS compounds (e.g., PFOS or PFOA) on specific tissues and organisms. This will allow scientists to identify better strategies for the prevention and/or mitigation of the molecular effects of PFASs. We also believe that the identification of the most conserved genetic responders to PFASs will support future research by providing new molecular venues of investigation for PFAS effects and also novel multi-species biomarkers, fueling the creation of ecosystem-wide tests for biological PFAS exposure.

## Figures and Tables

**Table 1:** Summary of the transcriptomics datasets analyzed in this study. PFOS: perfluorooctane sulfonic acid. PFOA: perfluorooctanoic acid. PFBS: perfluorobutanesulfonic acid. PFDS: perfluorodecanesulfonic acid. PFBA: perfluorobutanoic acid. PFPeA: perfluoropentanoic acid. PFHxA: perfluorohexanoic acid. PFHpA: perfluoroheptanoic acid. PFNA: perfluorononanoic acid. PFDA: perfluorodecanoic acid. PFUnA: perfluoroundecanoic acid. PFTeDA: perfluorotetradecanoic acid. PFHxS: perfluorohexanesulfonic acid. PFHpS: perfluoroheptanesulfonic acid. PFOSA: perfluorooctanesulfonamide. PAP: polyfluoroalkyl phosphate ester. FtS: fluorotelomer sulfonate. FtOH: fluorotelomer alcohol. HFPO-DA: hexafluoropropylene oxide-dimer acid. PFDoA: perfluorododecanoic acid.

Species	Sample Size	Platform	PFAS Compound	Concentration	Setup	Tissue	Reference
*H. sapiens*	607	RNA-seq	PFOS PFOA PFBS PFDS	0.02, 0.1, 0.2, 1, 2, 10, 20, 50, 100 µM	in vitro	Primary liver spheroids	Rowan-Carroll et al. 2021 [38]
*H. sapiens*	1201	RNA-seq	PFBA PFPeA PFHxA PFHpA PFOA PFNA PFDA PFUnA PFTeDA PFBS PFHxS PFHpS PFOS PFDS PFOSA 8:2MonoPAP 6:2MonoPAP 8:2 FtS 6:2 FtS 4:2 FtS 8:2 FtOH 6:2 FtOH 5:3 Acid	Various concentrations in the range 0.2–100 µM	in vitro	Primary liver spheroids	Reardon et al. 2021 [44]
*H. sapiens*	23	RNA-seq	PFOS	10 mg/kg	in vivo	Prostate cancer cells xenograft	Imir et al. 2021 [45]
*M. musculus*	32	RNA-seq	PFOA GenX	0.05, 0.3 mg/kg body weight/day	in vivo	Liver	Attema et al. 2022 [39]
*M. musculus*	37	RNA-seq	HFPO-DA	0.1, 0.5, 5 mg/kg	in vivo	Liver	Heintz et al. 2022 [46]
*M. musculus*	18	Microarray	PFOS PFNA	0.0003% of low-fat diet or high-fat diet	in vivo	Liver	Pfohl et al. 2021 [47]
*C. elegans*	60	RNA-seq	HFPO-DA	1.25 × 10^−5^, 6.25 × 10^−5^, 3.13 × 10^−4^, 1.56 × 10^−3^, 7.81 × 10^−3^, 1.56 × 10^−2^, 3.13 × 10^−2^, 6.25 × 10^−2^, 0.125, 0.25, 0.5, 1, 2, 4 g/L	in vivo	Whole body	Feng et al. 2022 [40]
*D. rerio*	16	RNA-seq	PFOSA	12.5 µM	in vivo	Embryo	Dasgupta et al. 2020 [41]
*G. morhua*	48	RNA-seq	PFOS PFOA PFNA	Low, medium, high, 1×, 20×, 100×	in vitro	Ovary	Khan et al. 2021 [43]
*M. salmoides*	72	Microarray	PFDA PFUnA PFDoA PFOS	Different for each lake and each PFAS	in vivo	Liver and Testis	Collí-Dulá et al. 2016 [48]
*P. promelas*	30	Microarray	PFOS PFBA PFHxA PFHpA PFOA PFNA PFDA	0.5, 25 µg/L	in vivo	Liver and Whole blood	Rodríguez-Jorquera et al. 2019 [49]

## Data Availability

All data used in this study is referenced (see Table 1) and publicly available in locations described in the Section 2.

## Supplementary Materials

The following supporting information can be downloaded at: https://www.mdpi.com/article/10.3390/toxics11070567/s1. Figure S1: correlation plot displaying the Pearson correlation coefficient between 110 PFAS vs. control contrasts across 11 datasets and 7 species. The color indicates the correlation coefficient, from the most negative (−1, dark blue) through no correlation (0, white) to the most positive (+1, dark red). The legend indicates the colors used to depict the eleven datasets. Figure S2: correlation plot showing the Pearson correlation coefficient between contrasts derived from M. musculus and H. sapiens datasets. The color indicates the correlation coefficient, from the most negative (−1, dark blue) through no correlation (0, white) to the most positive (+1, dark red). The legend indicates the colors used to depict the six datasets. Figure S3: scatter plot showing the positive correlation between two contrasts of *D. rerio* and *H. sapiens*. The highlighted and labeled genes are significantly (*p* < 0.05) and concordantly differentially expressed in response to PFAS exposure in both datasets. Figure S4: correlation plot showing the Pearson correlation coefficient between contrasts derived from fish species. The color indicates the correlation coefficient, from the most negative (−1, dark blue) through no correlation (0, white) to the most positive (+1, dark red). The legend indicates the colors used to depict the four datasets. Figure S5: line graph indicating the levels of expression of selected genes in response to PFAS molecules in different species, characterized by absolute integrated signature ≥ 10 and standard deviation ≥ 10. Each line is one gene: the genes shown here are the most consistently up- or down-regulated with high standard deviation, as extracted from the orange and cyan points of Figure 4. *x*-axis reports all the 110 contrasts analyzed in the integrated dataset, grouped by species. *y*-axis reports the signature for each gene, representing the significance (and sign) of the gene’s transcriptional response to PFAS. The horizontal lines delimit the *p*-value thresholds of 0.05. Figure S6. Line graphs showing the differential expression of selected genes across PFAS exposure: ACAT1 and UGT2A3 in panel A, and RPL35 and ESR1 in panel B. *y*-axis reports the signature for each gene, representing the significance (and sign) of the gene’s transcriptional response to PFAS, as −log10(*p*) × sign(log(FC)). The horizontal lines delimit the *p*-value thresholds of 0.05. Table S1: number and symbols of differentially expressed genes (at *p*-value ≤ 0.05) in all 110 PFAS versus control contrasts of the dataset. Table S2: table depicting pairwise inter-species orthologous conversions adopted in the current study. Table S3: table showing all signature values calculated in the combined dataset, where genes are shown as rows, and contrasts as columns. Abbreviations of each contrast are shown in the “Legend” Table S4: table showing the normalized enrichment scores for PFAS vs. control contrasts predicted for metabolites across the dataset. The contrast abbreviations follow the same Legend of Table S3.

## References

[1] Buck R.C., Franklin J., Berger U., Conder J.M., Cousins I.T., De Voogt P., Jensen A.A., Kannan K., Mabury S.A., Van Leeuwen S.P.J. (2011). Perfluoroalkyl and polyfluoroalkyl substances in the environment: Terminology, classification, and origins. Integr. Environ. Assess. Manag..

[2] Panieri E., Baralic K., Djukic-Cosic D., Buha Djordjevic A., Saso L. (2022). PFAS Molecules: A Major Concern for the Human Health and the Environment. Toxics.

[3] OECD (2021). Reconciling Terminology of the Universe of Per and Polyfluoroalkyl Substances: Recommendations and Practical Guidance.

[4] PubChem Classification Browser. https://pubchem.ncbi.nlm.nih.gov/classification/#hid=120.

[5] Gaines L.G.T., Sinclair G., Williams A.J. (2023). A proposed approach to defining per- and polyfluoroalkyl substances (PFAS) based on molecular structure and formula. Integr. Environ. Assess. Manag..

[6] CompTox Chemicals Dashboard. https://comptox.epa.gov/dashboard/chemical-lists/PFASSTRUCTV5.

[7] Organisation for Economic Co-operation and Development Toward a New Comprehensive Global Database of Per- and Polyfluoroalkyl Substances (PFASs): Summary Report on Updating the OECD 2007 List of Per- and Polyfluoroalkyl Substances (PFASs). https://www.oecd.org/officialdocuments/publicdisplaydocumentpdf/?cote=ENV-JM-MONO(2018)7&doclanguage=en.

[8] Gaines L.G.T. (2022). Historical and current usage of per- and polyfluoroalkyl substances (PFAS): A literature review. Am. J. Ind. Med..

[9] Podder A., Sadmani A.H.M.A., Reinhart D., Chang N.-B., Goel R. (2021). Per and poly-fluoroalkyl substances (PFAS) as a contaminant of emerging concern in surface water: A transboundary review of their occurrences and toxicity effects. J. Hazard. Mater..

[10] Piva E., Ioime P., Dall’Ara S., Fais P., Pascali J.P. (2022). Per- and polyfluoroalkyl substances (PFAS) determination in shellfish by liquid chromatography coupled to accurate mass spectrometry. Drug Test. Anal..

[11] Brase R.A., Mullin E.J., Spink D.C. (2021). Legacy and Emerging Per- and Polyfluoroalkyl Substances: Analytical Techniques, Environmental Fate, and Health Effects. Int. J. Mol. Sci..

[12] Beale D.J., Sinclair G.M., Shah R., Paten A.M., Kumar A., Long S.M., Vardy S., Jones O.A.H. (2022). A review of omics-based PFAS exposure studies reveals common biochemical response pathways. Sci. Total Environ..

[13] Fan L., Tang J., Zhang D., Ma M., Wang Y., Han Y. (2020). Investigations on the phytotoxicity of perfluorooctanoic acid in Arabidopsis thaliana. Environ. Sci. Pollut. Res. Int..

[14] Liu H., Hu W., Li X., Hu F., Liu Y., Xie T., Liu B., Xi Y., Su Z., Zhang C. (2022). Effects of perfluoroalkyl substances on root and rhizosphere bacteria: Phytotoxicity, phyto-microbial remediation, risk assessment. Chemosphere.

[15] Lucas K., Gaines L.G., Paris-Davila T., Nylander-French L.A. (2023). Occupational exposure and serum levels of per- and polyfluoroalkyl substances (PFAS): A review. Am. J. Ind. Med..

[16] Babayev M., Capozzi S.L., Miller P., McLaughlin K.R., Medina S.S., Byrne S., Zheng G., Salamova A. (2022). PFAS in drinking water and serum of the people of a southeast Alaska community: A pilot study. Environ. Pollut..

[17] LaKind J.S., Naiman J., Verner M.-A., Lévêque L., Fenton S. (2023). Per- and polyfluoroalkyl substances (PFAS) in breast milk and infant formula: A global issue. Environ. Res..

[18] LaKind J.S., Verner M.-A., Rogers R.D., Goeden H., Naiman D.Q., Marchitti S.A., Lehmann G.M., Hines E.P., Fenton S.E. (2022). Current Breast Milk PFAS Levels in the United States and Canada: After All This Time, Why Don’t We Know More?. Environ. Health Perspect..

[19] Lu Y., Meng L., Ma D., Cao H., Liang Y., Liu H., Wang Y., Jiang G. (2021). The occurrence of PFAS in human placenta and their binding abilities to human serum albumin and organic anion transporter 4. Environ. Pollut..

[20] Pascali J.P., Piva E., Bonasoni M.P., Migliavacca C., Seidenari A., Fais P. (2023). Analysis and distribution of per- and polyfluoroalkyl substances in decidua and villi placenta explants. Environ. Res..

[21] Piva E., Giorgetti A., Ioime P., Morini L., Freni F., Faro F.L., Pirani F., Montisci M., Fais P., Pascali J.P. (2021). Hair determination of Per- and polyfluoroalkyl substances (PFAS) in the Italian population. Toxicology.

[22] Cui Q., Pan Y., Wang J., Liu H., Yao B., Dai J. (2020). Exposure to per- and polyfluoroalkyl substances (PFASs) in serum versus semen and their association with male reproductive hormones. Environ. Pollut..

[23] Sunderland E.M., Hu X.C., Dassuncao C., Tokranov A.K., Wagner C.C., Allen J.G. (2019). A review of the pathways of human exposure to poly- and perfluoroalkyl substances (PFASs) and present understanding of health effects. J. Expo. Sci. Environ. Epidemiol..

[24] Fenton S.E., Ducatman A., Boobis A., DeWitt J.C., Lau C., Ng C., Smith J.S., Roberts S.M. (2021). Per- and Polyfluoroalkyl Substance Toxicity and Human Health Review: Current State of Knowledge and Strategies for Informing Future Research. Environ. Toxicol. Chem..

[25] Ehrlich V., Bil W., Vandebriel R., Granum B., Luijten M., Lindeman B., Grandjean P., Kaiser A.-M., Hauzenberger I., Hartmann C. (2023). Consideration of pathways for immunotoxicity of per- and polyfluoroalkyl substances (PFAS). Environ. Health.

[26] Ding N., Harlow S.D., Randolph J.F., Loch-Caruso R., Park S.K. (2020). Perfluoroalkyl and polyfluoroalkyl substances (PFAS) and their effects on the ovary. Hum. Reprod. Updat..

[27] Green M.P., Harvey A.J., Finger B.J., Tarulli G.A. (2021). Endocrine disrupting chemicals: Impacts on human fertility and fecundity during the peri-conception period. Environ. Res..

[28] Rickard B.P., Rizvi I., Fenton S.E. (2022). Per- and poly-fluoroalkyl substances (PFAS) and female reproductive outcomes: PFAS elimination, endocrine-mediated effects, and disease. Toxicology.

[29] Wang W., Hong X., Zhao F., Wu J., Wang B. (2023). The effects of perfluoroalkyl and polyfluoroalkyl substances on female fertility: A systematic review and meta-analysis. Environ. Res..

[30] Steenland K., Winquist A. (2021). PFAS and cancer, a scoping review of the epidemiologic evidence. Environ. Res..

[31] Girardi P., Merler E. (2019). A mortality study on male subjects exposed to polyfluoroalkyl acids with high internal dose of perfluorooctanoic acid. Environ. Res..

[32] Dunder L., Lind P.M., Salihovic S., Stubleski J., Kärrman A., Lind L. (2022). Changes in plasma levels of per- and polyfluoroalkyl substances (PFAS) are associated with changes in plasma lipids—A longitudinal study over 10 years. Environ. Res..

[33] Canova C., Barbieri G., Zare Jeddi M., Gion M., Fabricio A., Daprà F., Russo F., Fletcher T., Pitter G. (2020). Associations between perfluoroalkyl substances and lipid profile in a highly exposed young adult population in the Veneto Region. Environ. Int..

[34] Li Y., Barregard L., Xu Y., Scott K., Pineda D., Lindh C.H., Jakobsson K., Fletcher T. (2020). Associations between perfluoroalkyl substances and serum lipids in a Swedish adult population with contaminated drinking water. Environ. Health.

[35] Batzella E., Zare Jeddi M., Pitter G., Russo F., Fletcher T., Canova C. (2022). Associations between Mixture of Perfluoroalkyl Substances and Lipid Profile in a Highly Exposed Adult Community in the Veneto Region. Int. J. Environ. Res. Public Health.

[36] Rosen E.M., Kotlarz N., Knappe D.R.U., Lea C.S., Collier D.N., Richardson D.B., Hoppin J.A. (2022). Drinking Water–Associated PFAS and Fluoroethers and Lipid Outcomes in the GenX Exposure Study. Environ. Health Perspect..

[37] Roth K., Yang Z., Agarwal M., Liu W., Peng Z., Long Z., Birbeck J., Westrick J., Liu W., Petriello M.C. (2021). Exposure to a mixture of legacy, alternative, and replacement per- and polyfluoroalkyl substances (PFAS) results in sex-dependent modulation of cholesterol metabolism and liver injury. Environ. Int..

[38] Rowan-Carroll A., Reardon A., Leingartner K., Gagné R., Williams A., Meier M.J., Kuo B., Bourdon-Lacombe J., Moffat I., Carrier R. (2021). High-Throughput Transcriptomic Analysis of Human Primary Hepatocyte Spheroids Exposed to Per- and Polyfluoroalkyl Substances as a Platform for Relative Potency Characterization. Toxicol. Sci..

[39] Attema B., Janssen A.W.F., Rijkers D., van Schothorst E.M., Hooiveld G.J.E.J., Kersten S. (2022). Exposure to low-dose perfluorooctanoic acid promotes hepatic steatosis and disrupts the hepatic transcriptome in mice. Mol. Metab..

[40] Feng Z., McLamb F., Vu J.P., Gong S., Gersberg R.M., Bozinovic G. (2022). Physiological and transcriptomic effects of hexafluoropropylene oxide dimer acid in Caenorhabditis elegans during development. Ecotoxicol. Environ. Saf..

[41] Dasgupta S., Reddam A., Liu Z., Liu J., Volz D.C. (2020). High-content screening in zebrafish identifies perfluorooctanesulfonamide as a potent developmental toxicant. Environ. Pollut..

[42] Wolf C.J., Schmid J.E., Lau C., Abbott B.D. (2012). Activation of mouse and human peroxisome proliferator-activated receptor-alpha (PPARα) by perfluoroalkyl acids (PFAAs): Further investigation of C4–C12 compounds. Reprod. Toxicol..

[43] Khan E.A., Zhang X., Hanna E.M., Yadetie F., Jonassen I., Goksøyr A., Arukwe A. (2021). Application of quantitative transcriptomics in evaluating the ex vivo effects of per- and polyfluoroalkyl substances on Atlantic cod (*Gadus morhua*) ovarian physiology. Sci. Total. Environ..

[44] Reardon A.J.F., Rowan-Carroll A., Ferguson S.S., Leingartner K., Gagne R., Kuo B., Williams A., Lorusso L., Bourdon-Lacombe J.A., Carrier R. (2021). Potency Ranking of Per- and Polyfluoroalkyl Substances Using High-Throughput Transcriptomic Analysis of Human Liver Spheroids. Toxicol. Sci..

[45] Imir O.B., Kaminsky A.Z., Zuo Q.-Y., Liu Y.-J., Singh R., Spinella M.J., Irudayaraj J., Hu W.-Y., Prins G.S., Madak Erdogan Z. (2021). Per- and Polyfluoroalkyl Substance Exposure Combined with High-Fat Diet Supports Prostate Cancer Progression. Nutrients.

[46] Heintz M.M., Chappell G.A., Thompson C.M., Haws L.C. (2022). Evaluation of Transcriptomic Responses in Livers of Mice Exposed to the Short-Chain PFAS Compound HFPO-DA. Front. Toxicol..

[47] Pfohl M., Marques E., Auclair A., Barlock B., Jamwal R., Goedken M., Akhlaghi F., Slitt A.L. (2021). An Omics Approach to Unraveling the Paradoxical Effect of Diet on Perfluorooctanesulfonic Acid (PFOS) and Perfluorononanoic Acid (PFNA)-Induced Hepatic Steatosis. Toxicol. Sci..

[48] Collí-Dulá R.C., Martyniuk C.J., Streets S., Denslow N.D., Lehr R. (2016). Molecular impacts of perfluorinated chemicals (PFASs) in the liver and testis of male largemouth bass (*Micropterus salmoides*) in Minnesota Lakes. Comp. Biochem. Physiol. Part D Genom. Proteom..

[49] Rodriguez-Jorquera I.A., Colli-Dula R.C., Kroll K., Jayasinghe B.S., Parachu Marco M.V., Silva-Sanchez C., Toor G.S., Denslow N.D. (2019). Blood Transcriptomics Analysis of Fish Exposed to Perfluoro Alkyls Substances: Assessment of a Non-Lethal Sampling Technique for Advancing Aquatic Toxicology Research. Environ. Sci. Technol..

[50] Barrett T., Wilhite S.E., Ledoux P., Evangelista C., Kim I.F., Tomashevsky M., Marshall K.A., Phillippy K.H., Sherman P.M., Holko M. (2013). NCBI GEO: Archive for functional genomics data sets—Update. Nucleic Acids Res..

[51] Sayers E.W., Bolton E.E., Brister J.R., Canese K., Chan J., Comeau D.C., Farrell C.M., Feldgarden M., Fine A.M., Funk K. (2023). Database Resources of the National Center for Biotechnology Information in 2023. Nucleic Acids Res..

[52] Kim D., Paggi J.M., Park C., Bennett C., Salzberg S.L. (2019). Graph-based genome alignment and genotyping with HISAT2 and HISAT-genotype. Nat. Biotechnol..

[53] Liao Y., Smyth G.K., Shi W. (2014). feature Counts: An efficient general purpose program for assigning sequence reads to genomic features. Bioinformatics.

[54] Wickham H. (2016). Ggplot2.

[55] Mercatelli D., Lopez-Garcia G., Giorgi F.M. (2020). corto: A lightweight R package for gene network inference and master regulator analysis. Bioinformatics.

[56] Gu Z., Eils R., Schlesner M. (2016). Complex heatmaps reveal patterns and correlations in multidimensional genomic data. Bioinformatics.

[57] Carvalho B.S., Irizarry R.A. (2010). A framework for oligonucleotide microarray preprocessing. Bioinformatics.

[58] Davis S., Meltzer P.S. (2007). GEOquery: A bridge between the Gene Expression Omnibus (GEO) and BioConductor. Bioinformatics.

[59] Love M.I., Huber W., Anders S. (2014). Moderated estimation of fold change and dispersion for RNA-seq data with DESeq2. Genome Biol..

[60] Ritchie M.E., Phipson B., Wu D., Hu Y., Law C.W., Shi W., Smyth G.K. (2015). limma powers differential expression analyses for RNA-sequencing and microarray studies. Nucleic Acids Res..

[61] Alvarez M.J., Shen Y., Giorgi F.M., Lachmann A., Ding B.B., Ye B.H., Califano A. (2016). Functional characterization of somatic mutations in cancer using network-based inference of protein activity. Nat. Genet..

[62] Hu Y., Flockhart I., Vinayagam A., Bergwitz C., Berger B., Perrimon N., Mohr S.E. (2011). An integrative approach to ortholog prediction for disease-focused and other functional studies. BMC Bioinform..

[63] Schilder B. (2023). Orthogene: An R Package for Easy Mapping of Orthologous Genes across Hundreds of Species. https://bioconductor.org/packages/release/bioc/html/orthogene.html.

[64] Altschul S.F., Gish W., Miller W., Myers E.W., Lipman D.J. (1990). Basic local alignment search tool. J. Mol. Biol..

[65] Liberzon A., Subramanian A., Pinchback R., Thorvaldsdóttir H., Tamayo P., Mesirov J.P. (2011). Molecular signatures database (MSigDB) 3.0. Bioinformatics.

[66] Korotkevich G., Sukhov V., Sergushichev A. (2019). Fast Gene Set Enrichment Analysis. bioRxiv.

[67] Cavicchioli M.V., Santorsola M., Balboni N., Mercatelli D., Giorgi F.M. (2022). Prediction of Metabolic Profiles from Transcriptomics Data in Human Cancer Cell Lines. Int. J. Mol. Sci..

[68] Li H., Ning S., Ghandi M., Kryukov G.V., Gopal S., Deik A., Souza A., Pierce K., Keskula P., Hernandez D. (2019). The landscape of cancer cell line metabolism. Nat. Med..

[69] Anders S., Huber W. (2010). Differential expression analysis for sequence count data. Genome Biol..

[70] Powell P.K., Wolf I., Jin R., Lasker J.M. (1998). Metabolism of Arachidonic Acid to 20-Hydroxy-5,8,11, 14-Eicosatetraenoic Acid by P450 Enzymes in Human Liver: Involvement of CYP4F2 and CYP4A11. J. Pharmacol. Exp. Ther..

[71] Ni K.-D., Liu J.-Y. (2021). The Functions of Cytochrome P450 ω-hydroxylases and the Associated Eicosanoids in Inflammation-Related Diseases. Front. Pharmacol..

[72] Gao H., Cao Y., Xia H., Zhu X., Jin Y. (2020). CYP4A11 is involved in the development of nonalcoholic fatty liver disease via ROS-induced lipid peroxidation and inflammation. Int. J. Mol. Med..

[73] Wu Z., Ouyang T., Liu H., Cao L., Chen W. (2023). Perfluoroalkyl substance (PFAS) exposure and risk of nonalcoholic fatty liver disease in the elderly: Results from NHANES 2003–2014. Environ. Sci. Pollut. Res. Int..

[74] Glinos D.A., Garborcauskas G., Hoffman P., Ehsan N., Jiang L., Gokden A., Dai X., Aguet F., Brown K.L., Garimella K. (2022). Transcriptome variation in human tissues revealed by long-read sequencing. Nature.

[75] Chhibber A., French C.E., Yee S.W., Gamazon E.R., Theusch E., Qin X., Webb A., Papp A.C., Wang A., Simmons C.Q. (2017). Transcriptomic variation of pharmacogenes in multiple human tissues and lymphoblastoid cell lines. Pharm. J..

[76] Kim Y.-K., Kim Y.-S., Yoo K.-J., Lee H.-J., Lee D.-R., Yeo C.Y., Baek K.-H. (2007). The expression of Usp42 during embryogenesis and spermatogenesis in mouse. Gene Expr. Patterns.

[77] Calvert L., Green M.P., De Iuliis G.N., Dun M.D., Turner B.D., Clarke B.O., Eamens A.L., Roman S.D., Nixon B. (2021). Assessment of the Emerging Threat Posed by Perfluoroalkyl and Polyfluoroalkyl Substances to Male Reproduction in Humans. Front. Endocrinol..

[78] Subramanian A., Tamayo P., Mootha V.K., Mukherjee S., Ebert B.L., Gillette M.A., Paulovich A., Pomeroy S.L., Golub T.R., Lander E.S. (2005). Gene set enrichment analysis: A knowledge-based approach for interpreting genome-wide expression profiles. Proc. Natl. Acad. Sci. USA.

[79] Sen P., Qadri S., Luukkonen P.K., Ragnarsdottir O., McGlinchey A., Jäntti S., Juuti A., Arola J., Schlezinger J.J., Webster T.F. (2022). Exposure to environmental contaminants is associated with altered hepatic lipid metabolism in non-alcoholic fatty liver disease. J. Hepatol..

[80] Geiger S.D., Xiao J., Ducatman A., Frisbee S., Innes K., Shankar A. (2014). The association between PFOA, PFOS and serum lipid levels in adolescents. Chemosphere.

[81] Jin R., McConnell R., Catherine C., Xu S., Walker D.I., Stratakis N., Jones D.P., Miller G.W., Peng C., Conti D.V. (2020). Perfluoroalkyl substances and severity of nonalcoholic fatty liver in Children: An untargeted metabolomics approach. Environ. Int..

[82] Yang Z., Fu L., Cao M., Li F., Li J., Chen Z., Guo A., Zhong H., Li W., Liang Y. (2023). PFAS-induced lipidomic dysregulations and their associations with developmental toxicity in zebrafish embryos. Sci. Total Environ..

[83] Filatov M., Khramova Y., Parshina E., Bagaeva T., Semenova M. (2017). Influence of gonadotropins on ovarian follicle growth and development in vivo and in vitro. Zygote.

[84] Roepke T.A., Sadlier N.C. (2021). REPRODUCTIVE TOXICOLOGY: Impact of endocrine disruptors on neurons expressing GnRH or kisspeptin and pituitary gonadotropins. Reproduction.

[85] Cowland J.B., Borregaard N. (2016). Granulopoiesis and granules of human neutrophils. Immunol. Rev..

[86] Tang Z.-R., Zhang R., Lian Z.-X., Deng S.-L., Yu K. (2019). Estrogen-Receptor Expression and Function in Female Reproductive Disease. Cells.

[87] Stratakis N., Conti D.V., Jin R., Margetaki K., Valvi D., Siskos A.P., Maitre L., Garcia E., Varo N., Zhao Y. (2020). Prenatal Exposure to Perfluoroalkyl Substances Associated with Increased Susceptibility to Liver Injury in Children. Hepatology.

[88] Szilagyi J.T., Avula V., Fry R.C. (2020). Perfluoroalkyl Substances (PFAS) and Their Effects on the Placenta, Pregnancy, and Child Development: A Potential Mechanistic Role for Placental Peroxisome Proliferator–Activated Receptors (PPARs). Curr. Environ. Health Rep..

[89] Papadopoulou E., Stratakis N., Basagaña X., Brantsæter A.L., Casas M., Fossati S., Gražulevičienė R., Haug L.S., Heude B., Maitre L. (2021). Prenatal and postnatal exposure to PFAS and cardiometabolic factors and inflammation status in children from six European cohorts. Environ. Int..

[90] Gardener H., Sun Q., Grandjean P. (2021). PFAS concentration during pregnancy in relation to cardiometabolic health and birth outcomes. Environ. Res..

[91] Bloom M.S., Commodore S., Ferguson P.L., Neelon B., Pearce J.L., Baumer A., Newman R.B., Grobman W., Tita A., Roberts J. (2022). Association between gestational PFAS exposure and Children’s adiposity in a diverse population. Environ. Res..

[92] Hamilton K.J., Hewitt S.C., Arao Y., Korach K.S. (2017). Estrogen Hormone Biology. Curr. Top. Dev. Biol..

[93] Dupont S., Krust A., Gansmuller A., Dierich A., Chambon P., Mark M. (2000). Effect of single and compound knockouts of estrogen receptors α (ERα) and β (ERβ) on mouse reproductive phenotypes. Development.

[94] Rønnekleiv O.K., Qiu J., Kelly M.J. (2019). Arcuate Kisspeptin Neurons Coordinate Reproductive Activities with Metabolism. Semin. Reprod. Med..

[95] Gieske M.C., Kim H.J., Legan S.J., Koo Y., Krust A., Chambon P., Ko C. (2008). Pituitary Gonadotroph Estrogen Receptor-α Is Necessary for Fertility in Females. Endocrinology.

[96] Couse J.F., Bunch D.O., Lindzey J., Schomberg D.W., Korach K.S. (1999). Prevention of the Polycystic Ovarian Phenotype and Characterization of Ovulatory Capacity in the Estrogen Receptor-α Knockout Mouse. Endocrinology.

[97] Houck K.A., Patlewicz G., Richard A.M., Williams A.J., Shobair M.A., Smeltz M., Clifton M.S., Wetmore B., Medvedev A., Makarov S. (2021). Bioactivity profiling of per- and polyfluoroalkyl substances (PFAS) identifies potential toxicity pathways related to molecular structure. Toxicology.

[98] Zhao Z., Bo Z., Gong W., Guo Y. (2020). Inhibitor of Differentiation 1 (Id1) in Cancer and Cancer Therapy. Int. J. Med. Sci..

[99] Huang Y.-H., Hu J., Chen F., LeComte N., Basnet H., David C.J., Witkin M.D., Allen P.J., Leach S.D., Hollmann T.J. (2020). ID1 Mediates Escape from TGFβ Tumor Suppression in Pancreatic Cancer. Cancer Discov..

[100] Phelps D.W., Palekar A.I., Conley H.E., Ferrero G., Driggers J.H., Linder K.E., Kullman S.W., Reif D.M., Sheats M.K., DeWitt J.C. (2023). Legacy and emerging per- and polyfluoroalkyl substances suppress the neutrophil respiratory burst. J. Immunotoxicol..

[101] Grandjean P., Timmermann C.A.G., Kruse M., Nielsen F., Vinholt P.J., Boding L., Heilmann C., Mølbak K. (2020). Severity of COVID-19 at elevated exposure to perfluorinated alkylates. PLoS ONE.

[102] Nielsen C., Jöud A. (2021). Susceptibility to COVID-19 after High Exposure to Perfluoroalkyl Substances from Contaminated Drinking Water: An Ecological Study from Ronneby, Sweden. Int. J. Environ. Res. Public Health.

[103] Mercatelli D., Holding A.N., Giorgi F.M. (2021). Web tools to fight pandemics: The COVID-19 experience. Brief. Bioinform..

[104] Omoike O.E., Pack R.P., Mamudu H.M., Liu Y., Strasser S., Zheng S., Okoro J., Wang L. (2021). Association between per and polyfluoroalkyl substances and markers of inflammation and oxidative stress. Environ. Res..

[105] Barton K.E., Zell-Baran L.M., DeWitt J.C., Brindley S., McDonough C.A., Higgins C.P., Adgate J.L., Starling A.P. (2022). Cross-sectional associations between serum PFASs and inflammatory biomarkers in a population exposed to AFFF-contaminated drinking water. Int. J. Hyg. Environ. Health.

[106] Meneguzzi A., Fava C., Castelli M., Minuz P. (2021). Exposure to Perfluoroalkyl Chemicals and Cardiovascular Disease: Experimental and Epidemiological Evidence. Front. Endocrinol..

[107] Chen Z., Yang T., Walker D.I., Thomas D.C., Qiu C., Chatzi L., Alderete T.L., Kim J.S., Conti D.V., Breton C.V. (2020). Dysregulated lipid and fatty acid metabolism link perfluoroalkyl substances exposure and impaired glucose metabolism in young adults. Environ. Int..

[108] Fragki S., Dirven H., Fletcher T., Grasl-Kraupp B., Bjerve Gützkow K., Hoogenboom R., Kersten S., Lindeman B., Louisse J., Peijnenburg A. (2021). Systemic PFOS and PFOA exposure and disturbed lipid homeostasis in humans: What do we know and what not?. Crit. Rev. Toxicol..

[109] Forman H.J., Zhang H., Rinna A. (2009). Glutathione: Overview of its protective roles, measurement, and biosynthesis. Mol. Asp. Med..

[110] Dale K., Yadetie F., Müller M.B., Pampanin D.M., Gilabert A., Zhang X., Tairova Z., Haarr A., Lille-Langøy R., Lyche J.L. (2020). Proteomics and lipidomics analyses reveal modulation of lipid metabolism by perfluoroalkyl substances in liver of Atlantic cod (*Gadus morhua*). Aquat. Toxicol..

[111] Ojo A.F., Xia Q., Peng C., Ng J.C. (2021). Evaluation of the individual and combined toxicity of perfluoroalkyl substances to human liver cells using biomarkers of oxidative stress. Chemosphere.

[112] He L., Liu Y., Liu D., Feng Y., Yin J., Zhou X. (2021). Exogenous and Endogenous Serine Deficiency Exacerbates Hepatic Lipid Accumulation. Oxid. Med. Cell. Longev..

[113] Payne M., Stephens T., Lim K., Ball R.O., Pencharz P.B., Elango R. (2018). Lysine Requirements of Healthy Pregnant Women are Higher During Late Stages of Gestation Compared to Early Gestation. J. Nutr..

[114] Van Winkle L.J., Galat V., Iannaccone P.M. (2020). Lysine Deprivation during Maternal Consumption of Low-Protein Diets Could Adversely Affect Early Embryo Development and Health in Adulthood. Int. J. Environ. Res. Public Health.

[115] Schlett K. (2006). Glutamate as a Modulator of Embryonic and Adult Neurogenesis. Curr. Top. Med. Chem..

[116] Depeint F., Bruce W.R., Shangari N., Mehta R., O’Brien P.J. (2006). Mitochondrial function and toxicity: Role of the B vitamin family on mitochondrial energy metabolism. Chem. Biol. Interact..

[117] Wielsøe M., Long M., Ghisari M., Bonefeld-Jørgensen E.C. (2015). Perfluoroalkylated substances (PFAS) affect oxidative stress biomarkers in vitro. Chemosphere.

[118] Labine L.M., Oliveira Pereira E.A., Kleywegt S., Jobst K.J., Simpson A.J., Simpson M.J. (2022). Comparison of sub-lethal metabolic perturbations of select legacy and novel perfluorinated alkyl substances (PFAS) in *Daphnia magna*. Environ. Res..

