# Supplementary material for: Cross-Species Transcriptomics Analysis Highlights Conserved Molecular Responses to Per- and Polyfluoroalkyl Substances
Source: Toxics. 2023 Jun 29;11(7):567. doi: 10.3390/toxics11070567 (PMC10385990; doi:10.3390/toxics11070567)
Supplement: Supplementary file 1 [file toxics-11-00567-s001.zip › SupplementaryMaterials.docx]

Supplementary Materials


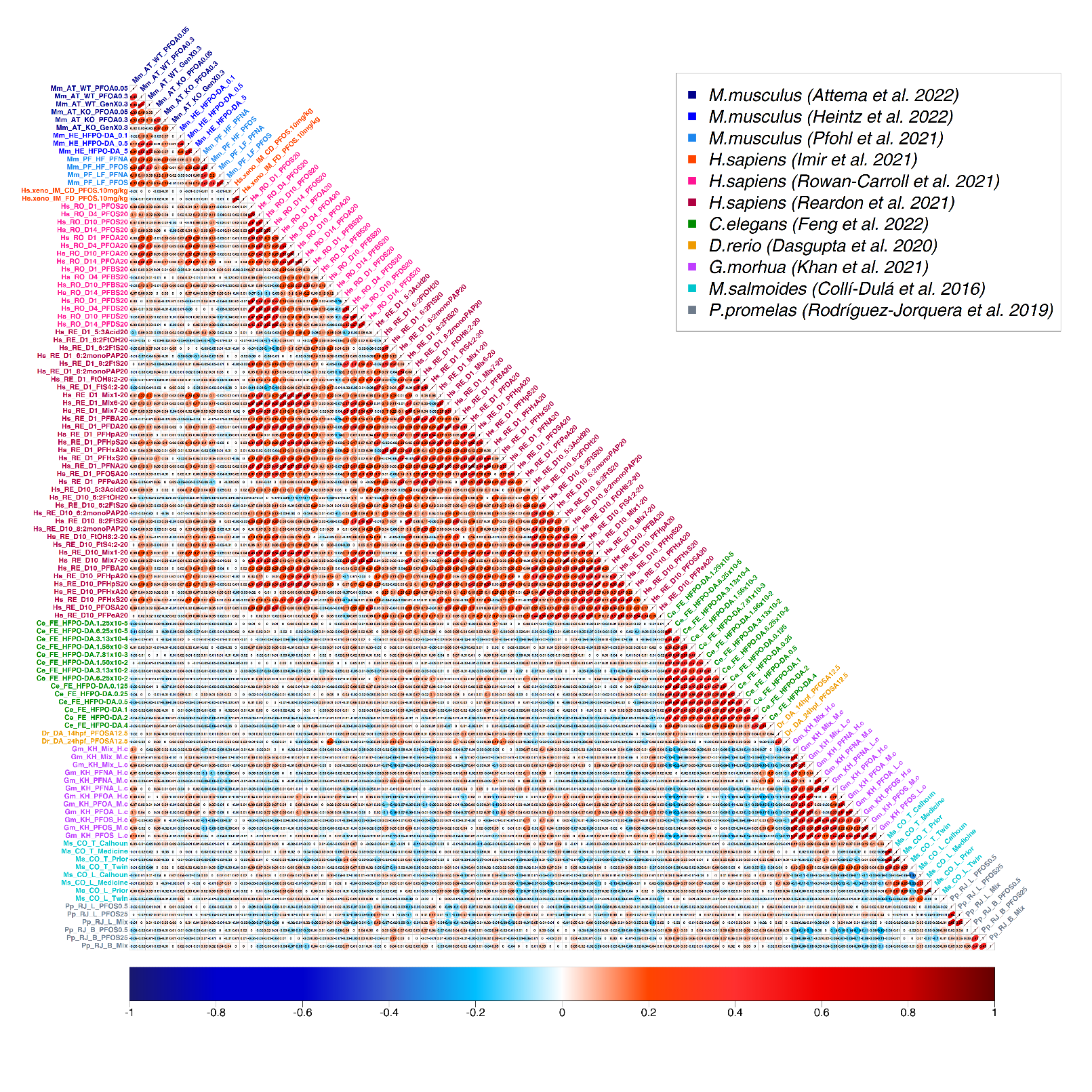


**Figure S1.** Correlation plot displaying the Pearson correlation coefficient between 110 PFAS vs. control contrasts across 11 datasets and 7 species. The color indicates the correlation coefficient, from the most negative (-1, dark blue) through no correlation (0, white) to the most positive (+1, dark red). The legend indicates the colors used to depict the eleven datasets.


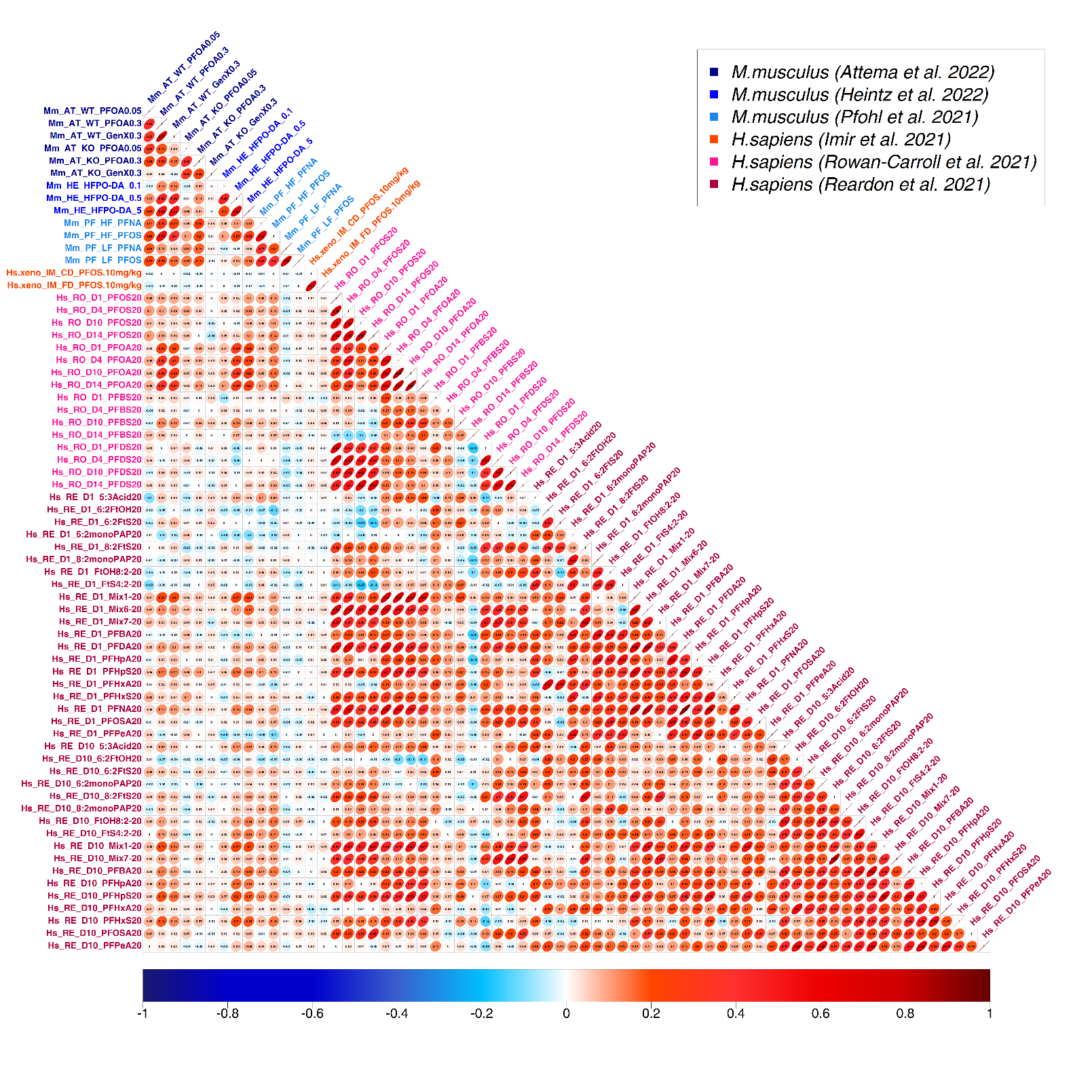


**Figure S2.** Correlation plot showing the Pearson correlation coefficient between contrasts derived from *M. musculus* and *H. sapiens* datasets. The color indicates the correlation coefficient, from the most negative (-1, dark blue) through no correlation (0, white) to the most positive (+1, dark red). The legend indicates the colors used to depict the six datasets.


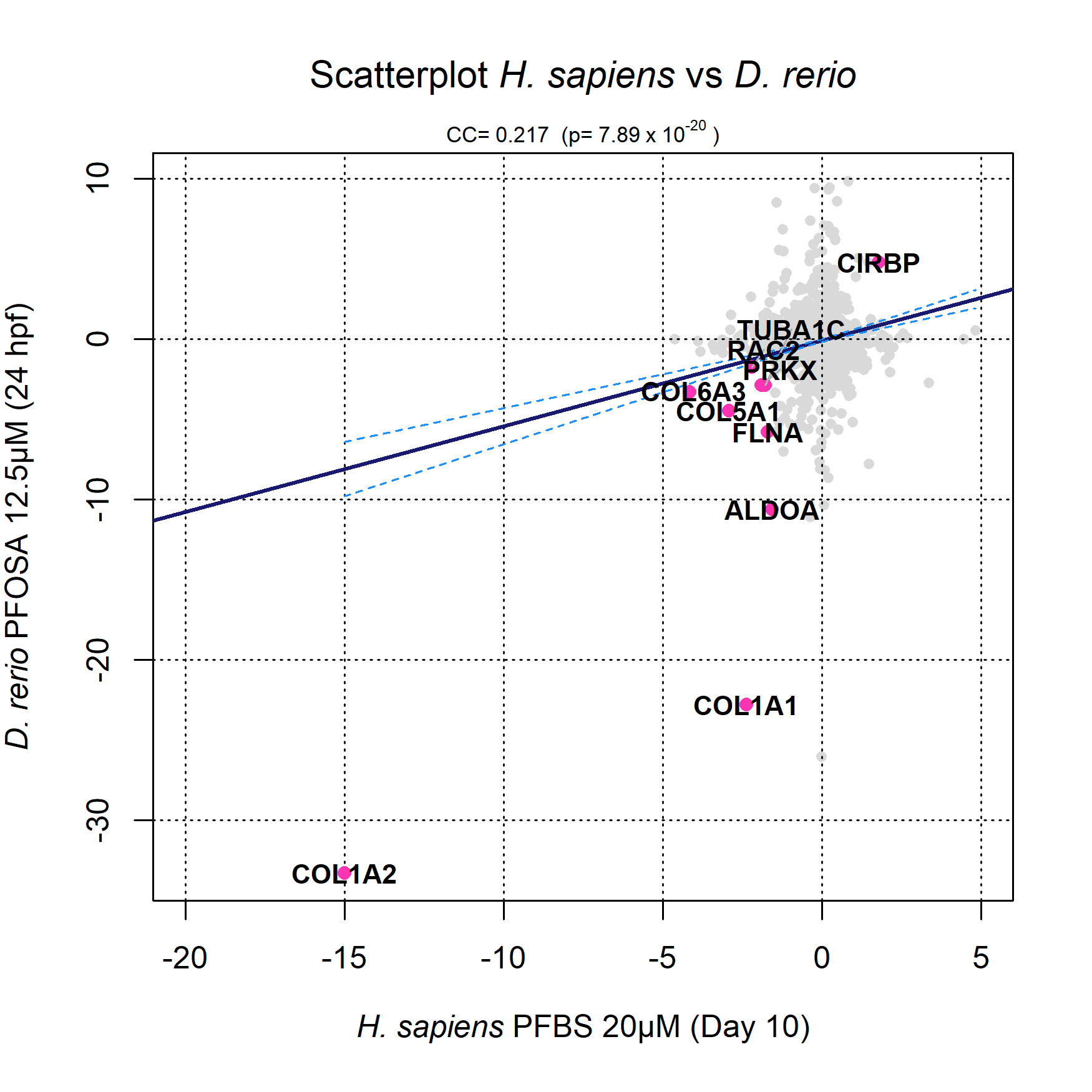


**Figure S3.** Scatter plot showing the positive correlation between two contrasts of *D. rerio* and *H. sapiens*. The highlighted and labeled genes are significantly (*p* <0.05) and concordantly differentially expressed in response to PFAS exposure in both datasets.


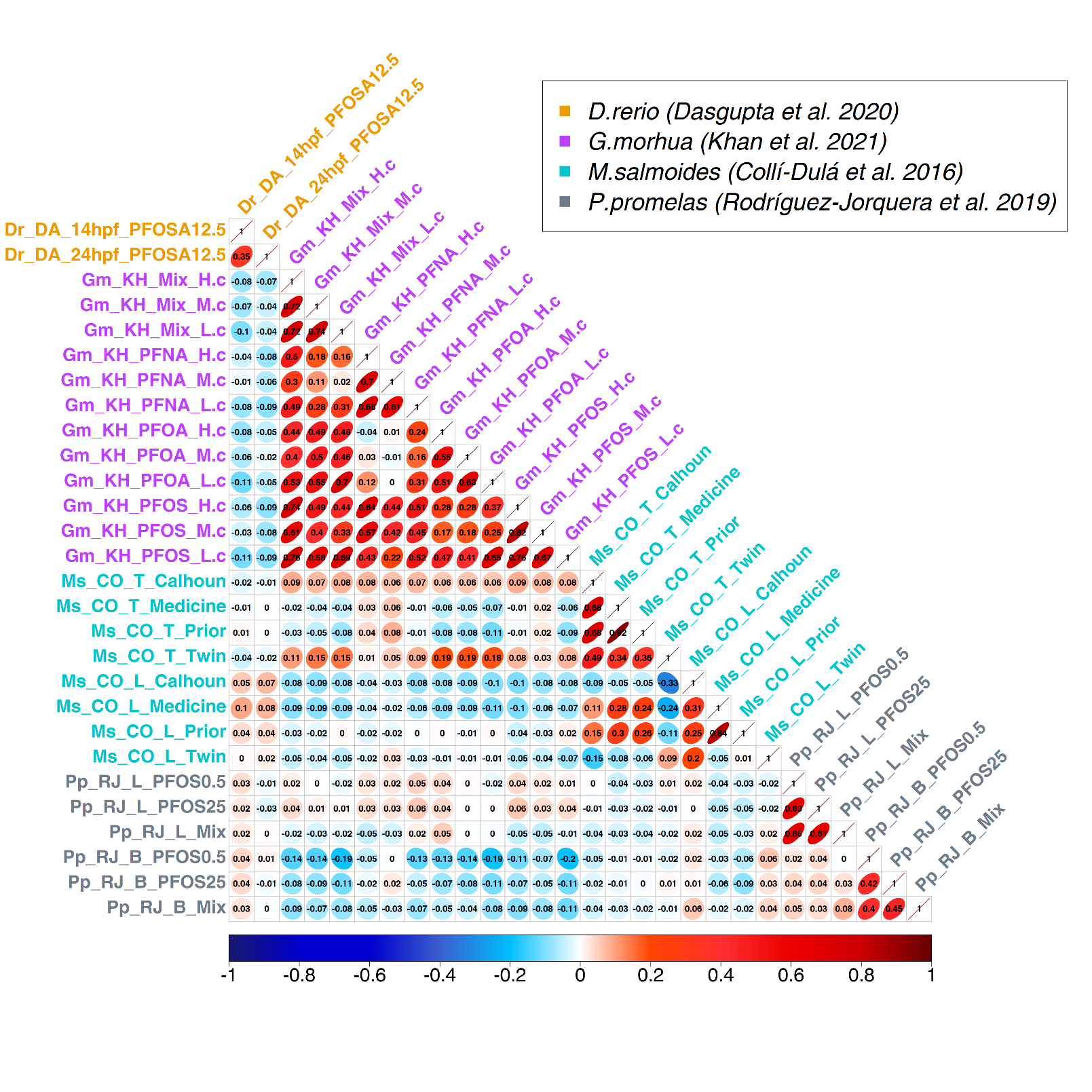


**Figure S4.** Correlation plot showing the Pearson correlation coefficient between contrasts derived from fish species. The color indicates the correlation coefficient, from the most negative (-1, dark blue) through no correlation (0, white) to the most positive (+1, dark red). The legend indicates the colors used to depict the four datasets.


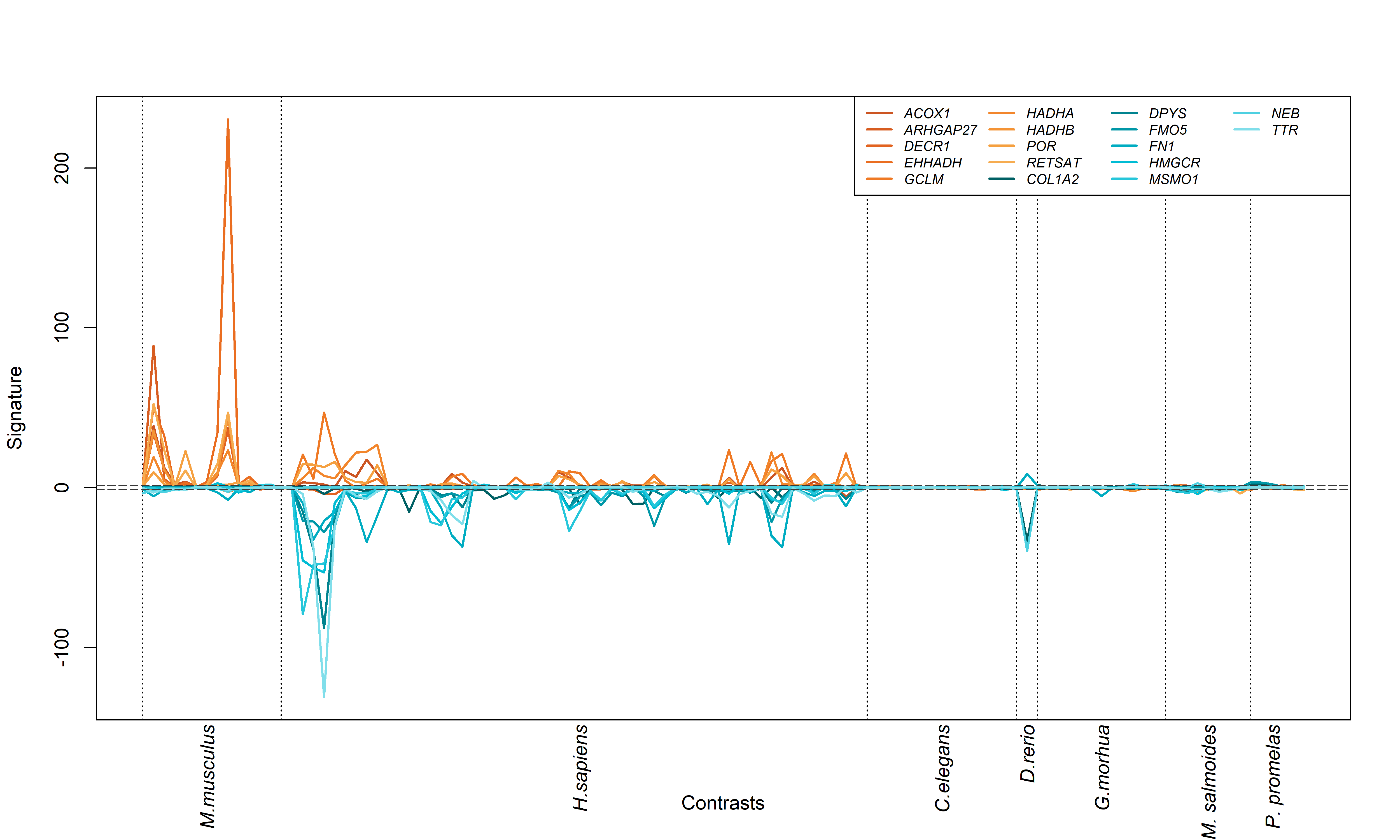


**Figure S5.** Line graph indicating the levels of expression of selected genes in response to PFAS molecules in different species, characterized by absolute integrated signature ≥ 10 and standard deviation ≥ 10. Each line is one gene: the genes shown here are the most consistently up- or down- regulated with high Standard deviation, as extracted from the orange and cyan points of Figure 4. X-axis reports all the 110 contrasts analyzed in the integrated dataset, grouped by species. Y-axis reports the signature for each gene, representing the significance (and sign) of the gene’s transcriptional response to PFAS. The horizontal lines delimit the p-value thresholds of 0.05.


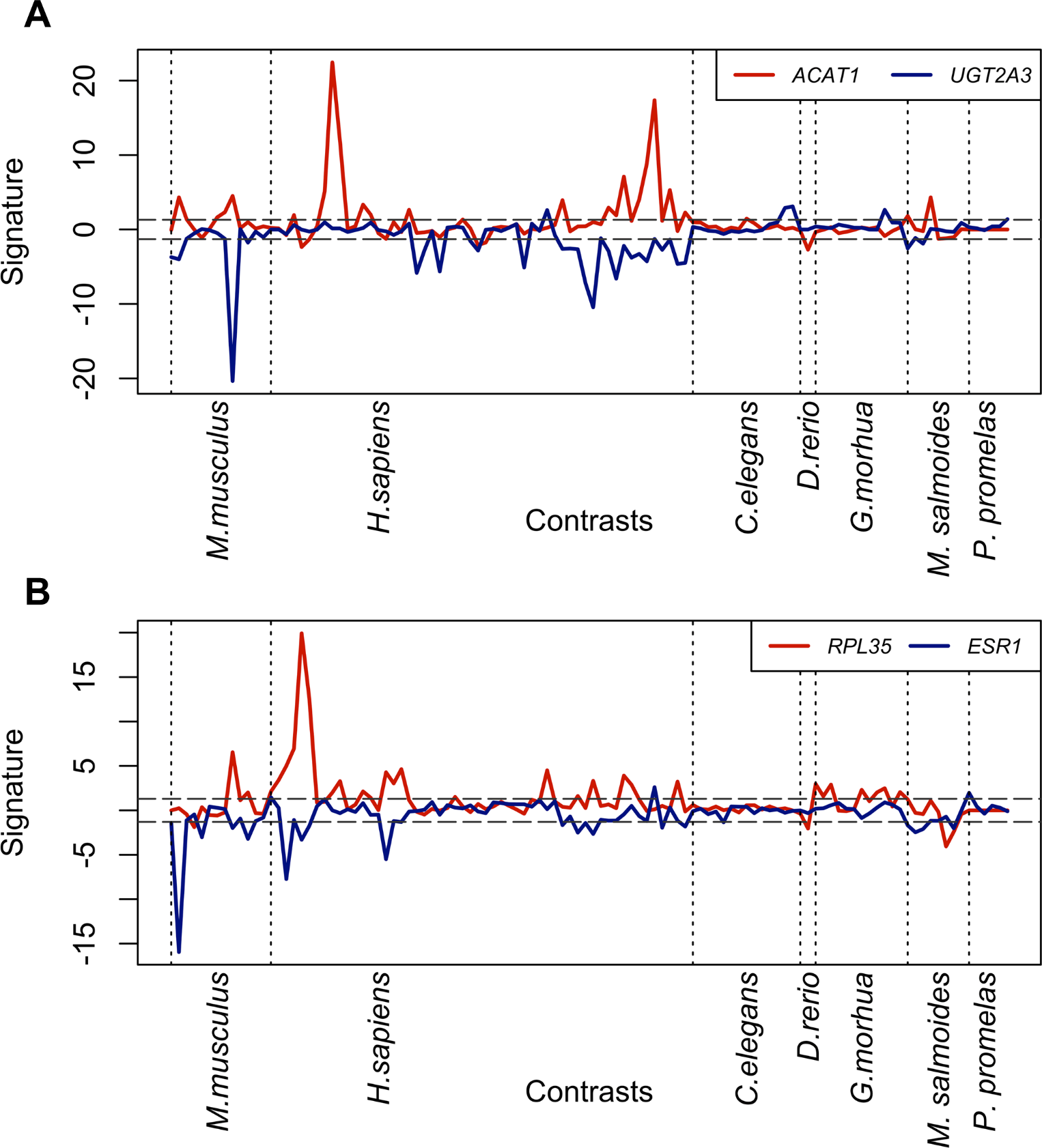


**Figure S6.** Line graphs showing the differential expression of selected genes across PFAS exposure: ACAT1 and UGT2A3 in panel A, and RPL35 and ESR1 in panel B. Y-axis reports the signature for each gene, representing the significance (and sign) of the gene’s transcriptional response to PFAS, as -log10(p) x sign(log(FC)). The horizontal lines delimit the p-value thresholds of 0.05.
